# Cross-Talk between the Cellular Redox State and the Circadian System in *Neurospora*

**DOI:** 10.1371/journal.pone.0028227

**Published:** 2011-12-02

**Authors:** Yusuke Yoshida, Hideo Iigusa, Niyan Wang, Kohji Hasunuma

**Affiliations:** Kihara Institute for Biological Research, Graduate School of Integrated Science, Yokohama City University, Totsuka-ku, Yokohama, Japan; Vanderbilt University, United States of America

## Abstract

The circadian system is composed of a number of feedback loops, and multiple feedback loops in the form of oscillators help to maintain stable rhythms. The filamentous fungus *Neurospora crassa* exhibits a circadian rhythm during asexual spore formation (conidiation banding) and has a major feedback loop that includes the FREQUENCY (FRQ)/WHITE COLLAR (WC) -1 and -2 oscillator (FWO). A mutation in *superoxide dismutase* (*sod*)*-1*, an antioxidant gene, causes a robust and stable circadian rhythm compared with that of wild-type (Wt). However, the mechanisms underlying the functions of reactive oxygen species (ROS) remain unknown. Here, we show that cellular ROS concentrations change in a circadian manner (ROS oscillation), and the amplitudes of ROS oscillation increase with each cycle and then become steady (ROS homeostasis). The ROS oscillation and homeostasis are produced by the ROS-destroying catalases (CATs) and ROS-generating NADPH oxidase (NOX). *cat-1* is also induced by illumination, and it reduces ROS levels. Although ROS oscillation persists in the absence of *frq*, *wc-1* or *wc-2*, its homeostasis is altered. Furthermore, genetic and biochemical evidence reveals that ROS concentration regulates the transcriptional function of WCC and a higher ROS concentration enhances conidiation banding. These findings suggest that the circadian system engages in cross-talk with the cellular redox state via ROS-regulatory factors.

## Introduction

Biological processes, such as development and physiology are regulated via underlying circadian clock mechanisms that are synchronized with the daily light cycle of the Earth. The circadian rhythm (“circa”: around; “diem: day) is composed of self-sustaining oscillations with a period of approximately 24 hr [1]. The circadian clock is composed of three elements: an input pathway, an oscillator and an output pathway. The main oscillator in circadian clocks is likely a transcription-translation feedback loop (TTFL) that is present in a diverse range of organisms from bacteria to humans [2]. A portion of the positive feedback mechanism in eukaryotes consists of transcription factors that form heterodimers via PER, ARNT and SIM (PAS) domains [3]. The input pathway resets the oscillator based on daily changes to external stimuli (entrainments), such as light and temperature; the oscillator can be entrained to periods around 24 hr. The oscillator then controls circadian rhythmic behaviors (various biological activities and aspects of physiology) via the output pathway [2]. Multiple feedback loops, not a single isolated loop, must be linked together to sustain a stable rhythm [4].

The filamentous fungus *Neurospora crassa* exhibits a circadian rhythm during asexual spore formation (conidiation). The rhythm has a period of approximately 22 hr in continuous darkness and this model organism is commonly used for studying circadian clock biology. *Neurospora*'s oscillator is composed of the FRQ (FREQUENCY) and WC (WHITE COLLAR)-1 and WC-2 complex (WCC) and called FRQ/WCC oscillator (FWO). WC-1 and WC-2 formed a heterodimer via the PAS domain. The mechanisms underlying the function of FWO are well understood and comprise a TTFL. WCC, a transcription factor, binds to the Clock box (C-box) in the *frq* promoter region and promotes *frq* expression [5], [6]. The *frq* transcript oscillates in a circadian manner, and the levels reach a maximum at circadian time (CT) 4 and trough a minimum at CT 16 [7]. However, many studies have shown that *frq*-null mutants exhibit rhythmic conidiation under particular conditions such as the addition of farnesol and menadione to the medium as well as in *chain elongation* (*cel*), *vivid* (*vvd*) or *sod-1* double mutants [8]–[11]. These data indicate the existence of other oscillators, such as FRQ-less oscillator (FLO) and WC-FLO [12]–[15]. Although the molecular mechanisms underlying the additional oscillators and their relationships with each other remain unknown, several observations have suggested the presence of a larger and more complex regulatory system [14]. These oscillators regulate the expression of clock-controlled genes (*ccgs*) via output pathways, including the osmotically sensitive (OS) MAPK pathway, to facilitate rhythmic conidiation [16].

Although the free-running rhythm in *Neurospora* has a period length of approximately 22 hr, the rhythm can be entrained to a daily (24 hr) cycle via light and temperature. WC-1 possesses a light-oxygen-voltage (LOV) domain, which is a type of the PAS domain and can bind flavin adenine dinucleotide (FAD) via LOV. Binding to the C-box by WCC is enhanced by illumination and induces *frq* expression [5], [17]. VIVID (VVD) is also a flavin-binding photo-receptor with a LOV domain, similar to WC-1 [18], [19]. VVD is considered a negative regulator of WCC, preventing the transcriptional activation of WCC [20]. WCC also functions as a photoreceptor in the circadian clock and during light-inducible carotenoid synthesis [17], [21]. Previously, we demonstrated the function of WCC accelerated light-induced carotenoid synthesis in both *sod-1*-deficient and ROS-treated cells [22], [23].

ROS include singlet oxygen, superoxide (O_2_^−^), hydrogen peroxide (H_2_O_2_) and hydroxyl radicals. Cellular ROS are passively generated by the mitochondria via respiration, as well as metabolism, and are actively generated by NADPH oxidase (NOX) [24]. Excess ROS are rapidly detoxified by ROS scavengers. In many organisms, singlet oxygen is removed by antioxidants such as carotenoids; O_2_^−^ is reduced to H_2_O_2_ by SOD; and H_2_O_2_ is reduced to water by catalase (CAT). Our observations suggested that cellular ROS affect WCC-mediated carotenogenesis. Because WCC is a photoreceptor, transcription factor, and a positive element in the circadian clock, we examined whether ROS affect circadian rhythms. *Neurospora* with *sod-1* mutations demonstrate robust conidial banding compared with the wild type (Wt) and are hyper-sensitive to light entrainment [10], [25]. Moreover, rhythmic conidiation is observed in *Neurospora* with both *sod-1* and *frq* mutations [10], which suggests that cellular ROS modulate circadian output via FWO and FLO and stabilize conidial banding. In this study, we measured cellular ROS levels during conidiation banding to examine the behavior of cellular ROS, and we observed circadian oscillations of intracellular ROS during growth. We then investigated the role of cellular ROS in the circadian system and attempted to identify the mechanisms that manage the cellular ROS level.

## Results

### Cellular ROS profiles in *Neurospora*

**Figure 1:**
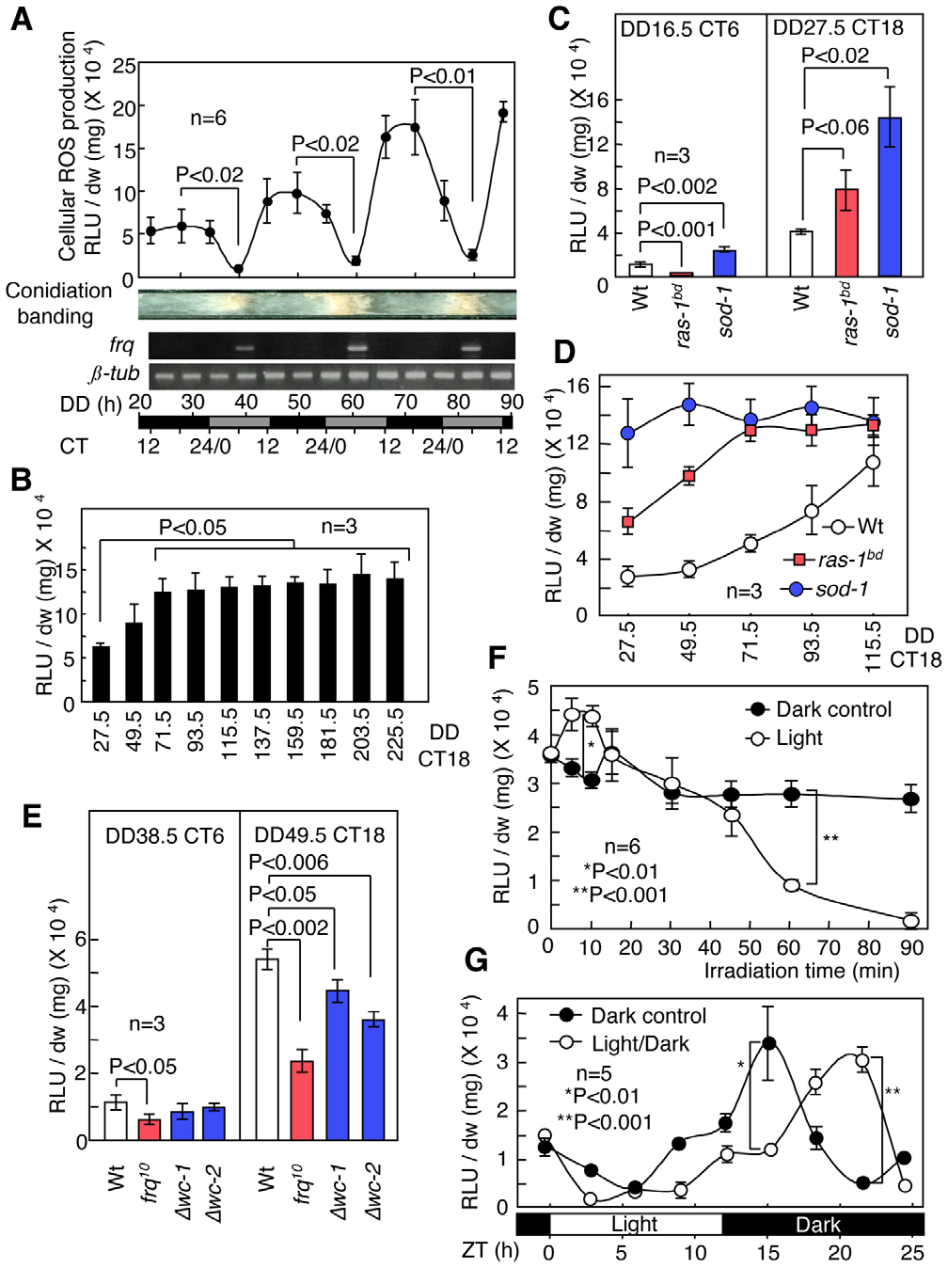
Cellular ROS profiles in *Neurospora*. (A) Temporal profiles of cellular ROS levels under constant darkness (DD) in *ras-1^bd^* mutants. Cellular ROS levels in the growth fronts from the race tube were measured using a lucigenin chemiluminescence assay. *frq* and *ß-tub* transcripts were detected using semi-quantitative RT-PCR and were used as experimental controls. (B) Cellular ROS generation at CT 18 in subsequent cycles for *ras-1^bd^* mutants. Mycelia were harvested at CT 18 during each cycle (from DD 27.5 to 225.5). (C) ROS generation in mycelia at the minimum (CT 6) and maximum levels (CT 18) for the Wt as well as the *ras-1^bd^* and *sod-1* mutants. (D) Cellular ROS generation in mycelia at CT 18 in subsequent cycles for the Wt as well as the *ras-1^bd^* and *sod-1* mutants. (E) Cellular ROS generation in mycelia for the Wt and the clock mutants (*frq^10^*, *Δwc-1* and *Δwc-2*) at CT 6 and 18. (F) Effect of brief light exposure on cellular ROS levels. Mycelia in the race tube were exposed to light at CT 18 (DD 27.5) for 5, 10, 15, 30, 45, 60 and 90 min. (G) Effects of the LD cycle on cellular ROS levels. Mycelia in the race tube were exposed to light for 12 hr at CT 24 (DD 11) and then transferred to dark conditions. Cellular ROS levels were measured in 3-h intervals. CT = circadian time, ZT = Zeitgeber time. All values are shown as mean ± standard error (SEM).

To examine the cellular ROS behavior during the daily cycle, we directly harvested the growth front in the race tube and measured the level of intracellular ROS because cellular ROS concentrations depend on the culture conditions (see Materials and Methods, Fig. S1). The liquid culture assay is the conventional method used to analyze molecular profiles in *Neurospora* clock studies. We checked *frq* rhythmic expression as methodological control. To measure cellular ROS levels, we used a lucigenin chemiluminescence assay, the results of which were supported using additional assays. The lucigenin chemiluminescence assay was primarily used to detect ROS generation. In general, this assay detects superoxide radicals in a cell [26]. However, H_2_O_2_ can also be detected depending on the conditions (Fig. S2) [27]. Hence, the lucigenin assay detects superoxide radicals and H_2_O_2_. We used a *band* (*bd*) mutant that exhibits clear conidial banding and has been used as a wild type in *Neurospora* circadian clock studies [28] to first measure ROS levels. The *bd* mutation is a point mutation in *ras-1*, and we indicate the *bd* mutants as *ras-1^bd^* in this paper [25]. The cellular ROS concentration in *ras-1^bd^* mutants displayed a circadian oscillation with a period length of approximately 22 hr; the amplitude of this oscillation gradually increased with each cycle and stabilized after 3 cycles (Fig. 1A, B and Fig. S3, S4, S5). The peak corresponded to CT 12–18, and the trough corresponded to CT 6.

To confirm higher ROS levels in the *sod-1* mutants during conidiation banding, we assessed cellular ROS levels at the minimum (CT 6) and maximum (CT 18) points during the ROS oscillation. During the initial growth, both the *sod-1* and the *ras-1^bd^* mutants displayed ROS oscillations with a higher amplitude than those of the Wt (Fig. 1C). However, each maximum level in the *sod-1* and *ras-1^bd^* mutants converged to a constant level after 3 cycles (Fig. 1D). The results obtained from *ras-1^bd^* suggest that RAS signaling is involved in cellular ROS generation. A standard Wt strain was used as a background strain in subsequent experiments to minimize the complexity of the results. We also examined temporal ROS levels in the *frq^10^*, *Δwc-1* and *Δwc-2* mutants, which are arrhythmic mutants, to determine whether ROS oscillation requires a circadian clock. Although *frq^10^*, *Δwc-1* and *Δwc-2* mutants demonstrated ROS oscillation, the ROS levels in the *frq^10^* at CT 6 and 18 were half of those of the Wt, and the amplitudes in *Δwc-1* and *Δwc-2* were slightly weaker than in the Wt (Fig. 1E).

Environmental cues, such as light and temperature, reset the circadian rhythm to a period of approximately 24 hr. In fish and flies, the cellular ROS level depends on the presence of light and varies with the light/dark (LD) cycle [29], [30]. In *Neurospora*, the cellular ROS levels in the mycelium were reduced by light at CT 18, but not by temperature changes (Fig. S6). The ROS levels transiently increased after 5–10 min of illumination and then decreased after 60 min of illumination (Fig. 1F). The transient induction of ROS may be generated from intracellular photo-sensitizers, such as flavin [31]. Under photoperiodic conditions, continuous light attenuated the increase in ROS levels. ROS levels increased again after approximately 10 hr of illumination but then underwent a phase shift when the sample was placed in the dark (Fig. 1G). These results suggest that cellular ROS levels are controlled by circadian clocks and illumination.

### Cellular ROS levels are managed via circadian clocks using catalase

**Figure 2:**
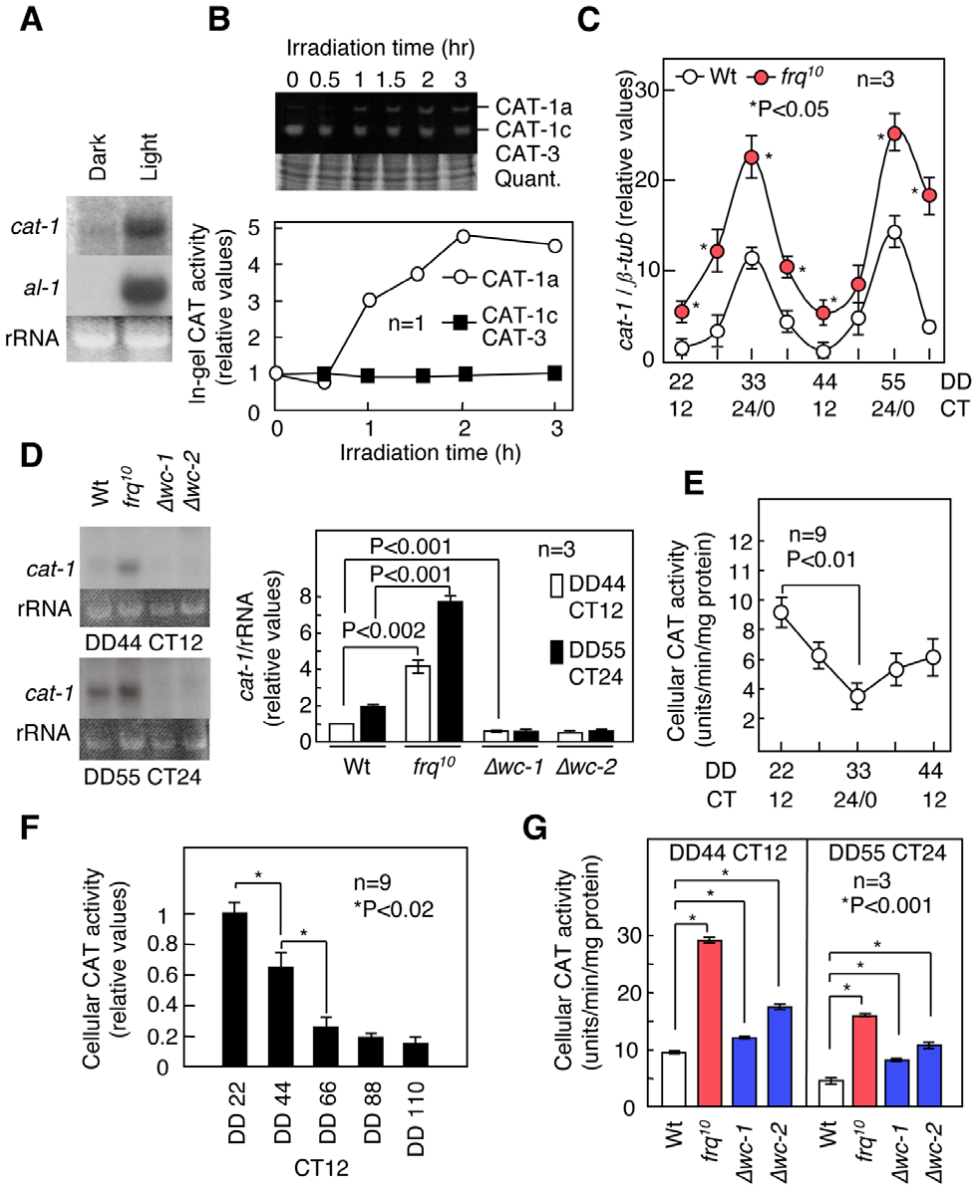
The control of catalase via light and circadian clocks. (A) Light-induced *cat-1* expression. The *cat-1* transcripts in mycelia at CT 18 (DD 49.5) after 1 h of illumination were detected using northern blot analysis. The expression of *albino* (*al*)*-1* served as a positive control. (B) Catalase activity during illumination, as measured using an in-gel assay. Mycelia were exposed to light at CT 18 (DD 49.5). CAT-1 was detected as CAT-1a (reduced form) and CAT-1c (oxidized form) because of the oxidative conditions in catalases. (C) Temporal *cat-1* expression in the race tube growth fronts from the Wt and *frq^10^* mutants as determined by qRT-PCR. (D) *cat-1* mRNA accumulation in the race tube growth fronts from the Wt, *frq^10^*, *Δwc-1* and *Δwc-2* mutants at CT 12 (minimum) and 24 (maximum), as assayed by northern blot analysis. (E) Temporal cellular catalase activity in Wt cells. Cellular catalase activity was determined using a spectrometric assay. (F) Cellular catalase activity at CT 12 (maximum) in subsequent cycles for the Wt, as measured by a spectrometric assay. (G) Cellular catalase activity at CT 12 (maximum) and 24 (minimum) in the Wt and *frq^10^*, as well as the *Δwc-1* and *Δwc-2* mutants, as measured by a spectrometric assay. All values, more than three replicates, are shown as mean ± standard error (SEM).

To understand how cellular ROS levels are managed, we next examined the cellular antioxidants present in cells exposed to light. In *Neurospora*, of the four CAT genes (CAT-1, CAT-2, CAT-3 and CCT-1/CAT-4), CAT-1 is the only isoform that is light-inducible [32], [33]. Cellular CAT activity increased slightly after illumination (Fig. S7), especially CAT-1 activity, and *cat-1* transcripts were significantly induced at CT 18 when the mycelia were exposed to light (Fig. 2A & B). Furthermore, we speculated that the ROS oscillation observed under free-running conditions might also be produced by CAT-1. To test this, we evaluated *cat-1* mRNA levels using a quantitative real-time PCR (qRT-PCR) assay. *cat-1* transcripts from Wt cells oscillated with a peak at CT 24/0; the amplitude of the *cat-1* oscillation in the *frq^10^* mutants was higher than that in the Wt (Fig. 2C). Furthermore, the *cat-1* transcripts from the *Δwc-1* and *Δwc-2* mutants were arrhythmic and much lower compared with the Wt (Fig. 2D). These results suggest that *wc-1* and *wc-2* up-regulate the expression of *cat-1*. Cellular CAT activity also oscillated slightly with a peak at CT 12 (Fig. 2E). Interestingly, the phase for cellular CAT activity was out of sync with the phase for the *cat-1* transcripts. This result suggests that CAT family members other than CAT-1 may mask CAT-1 activity after cell lysis. Cellular CAT activity levels at CT 12, the maximum during the CAT activity oscillation, decreased with each cycle and then stabilized at lower levels (Fig. 2F). The increase and ensuing stability of the maximum ROS levels during each cycle may result from a decrease in cellular CAT during each cycle. In *frq^10^* mutants, the CAT activity levels were higher than in the Wt, which is consistent with the changes in ROS levels and *cat-1* expression (Fig. 2G). However, the *cat-1* levels in the *Δwc-1* and *Δwc-2* mutants were not consistent with the results from testing cellular catalase activity and cellular ROS levels (Fig. 2G). These observations suggest that additional CATs may supplement the cellular peroxidative capacity when *cat-1* transcripts become low in the absence of *wc-1* and *wc-2*.

**Figure 3:**
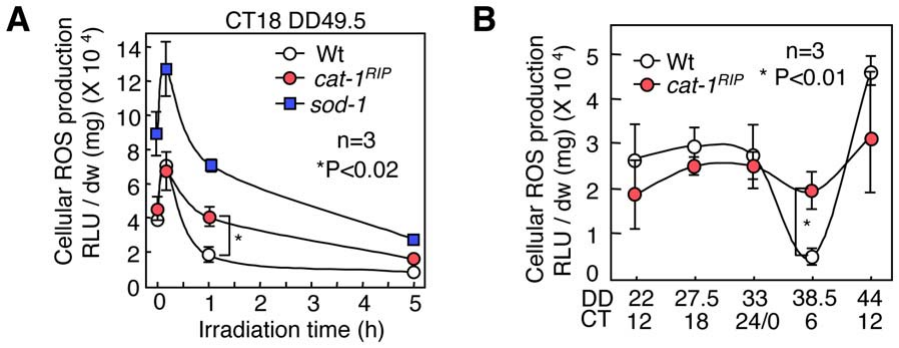
Determination of the roles of *cat-1* in cellular ROS using a *cat-1* loss-of-function mutant. (A) Effect of light on cellular ROS levels in *cat-1^RIP^* and *sod-1* mutants. Mycelia in the race tube growth fronts were exposed to light for 10 min, 1 hr and 5 hr at CT 18. Cellular ROS levels were measured using a lucigenin chemiluminescence assay. (B) Daily ROS generation from Wt and *cat-1^RIP^*cells. All values are shown as mean ± standard error (SEM).

To examine whether the ROS-destroying function of CAT affects cellular ROS levels and the circadian rhythm, we inhibited all CATs using 3-AT (3-amino-1,2,4-triazole). Wild-type samples that were treated with 3-AT displayed a robust conidial banding, similar to that of the *sod-1* mutant, and high ROS levels (Fig. S8). These results indicated that cellular CATs are involved in the conidial banding and cellular ROS levels. We also isolated a *cat-1* loss-of-function mutant (*cat-1^RIP^*) to examine the specific effects of CAT-1 [34]. The transient increase in ROS after illumination was similar in Wt and *cat-1^RIP^*cells, although the ROS levels in *cat-1^RIP^* were slightly reduced compared with those of the Wt and decreased more slowly (Fig. 3A). In addition to CAT-1, mycelial carotenoids are induced by light and may eliminate cellular ROS [35]. Thus, the induction of carotenoids rather than CAT-1 could reduce the ROS levels. ROS levels were significantly higher in the *sod-1* mutants (Fig. 3A). With respect to the cellular ROS oscillation, the levels in *cat-1^RIP^* at CT 6 were higher than those in the Wt, and the oscillations were moderate compared with those of the Wt (Fig. 3B). The maximum ROS levels at CT 18 for each subsequent cycle were not significantly different between Wt and *cat-1^RIP^* cells (Fig. S9). This result suggested that *cat-1* is involved in ROS oscillation but not in homeostasis. Conidial banding and *frq* expression in *cat-1^RIP^* was normal in DD after light-resetting and light-entrainment (LD) (Fig. S10, S11, S12). These results indicate that the lack of ROS oscillation does not affect the circadian rhythms.

### Cellular ROS are mainly produced by NADPH oxidase

**Figure 4:**
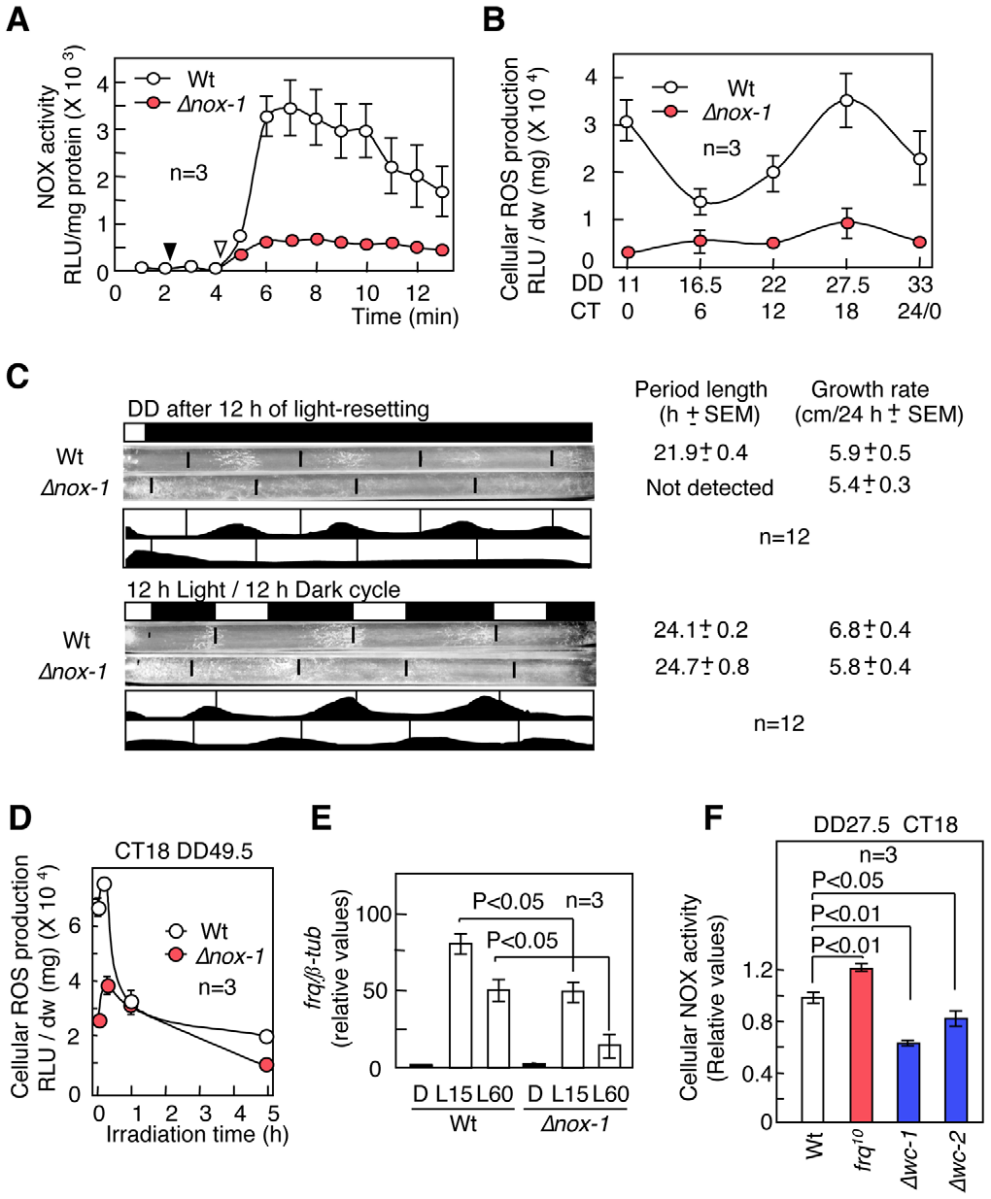
Generation of cellular ROS by NADPH oxidase (NOX). (A) Cellular NOX activity in the Wt and *Δnox-1* mutants. Mycelia harvested at CT 18 were disrupted and used as cell lysates in the assay. Cell lysates and NADPH were added to the assay buffer at the points noted using closed and open arrows, respectively. Temporal ROS generation (RLU/mg protein) by NOX was detected using lucigenin chemiluminescence. (B) Daily ROS generation in the mycelia of the Wt and *Δnox-1* as assayed by the lucigenin chemiluminescence assay. (C) Conidiation banding in *Δnox-1*. Conidial banding in the Wt and *Δnox-1* in the dark after 12 hr light of light-resetting (upper) and a 12 hr light/-12 hr dark cycle (lower). (D) Effect of light on cellular ROS levels in the Wt and *Δnox-1*. (E) Light-induced *frq* mRNA accumulation in the Wt and *Δnox-1*. Mycelia in the race tube growth fronts at CT 24/0 (DD 11 h) were exposed to light for 15 min (L15) and 60 min (L60). *frq* transcripts were detected using qRT-PCR. (F) Cellular NOX activity (measured as RLU/min/mg protein) at CT 18 in the Wt, *frq^10^*, and the *Δwc-1* and *Δwc-2* mutants. All values are shown as mean ± standard error (SEM).

We examined NADPH oxidase (NOX), an active ROS generator. In *Neurospora*, two NOXs (NOX-1 and NOX-2) have been isolated [24]. *Δnox-1* mutants had a greater reduction in cellular NOX activity, low ROS levels and a loss of cellular ROS oscillation (Fig. 4A, B and Fig. S13). For conidiation banding, *Δnox-1* mutants displayed an arrhythmic phenotype under free-running conditions and indeterminate conidiation in an LD cycle compared to the Wt (Fig. 4C, Fig. S14 and S15). The expression of light-inducible genes, such as *frq* and *albino*s (*al*s), was elevated in *sod-1* mutants compared with in the Wt [10], [22]. In contrast, lower ROS levels may reduce the level of light-induced *frq* transcripts because cellular ROS levels in the *Δnox-1* mutants were low during the transient ROS increase (Fig. 4D). As we expected, the light-induced *frq* level in the *Δnox-1* mutants was lower than in the Wt (Fig. 4E). Cellular NOX activity showed no circadian rhythmicity: it slightly decreased during the initial growth and then subsequently stabilized (Fig. S16). NOX activity was slightly altered in the *frq^10^*, *Δwc-1* and *wc-2* mutants (Fig. 4F). These results suggest that NOX can be regulated by the circadian system to generate cellular ROS.

### Redox state can affect the function of WCC

**Figure 5:**
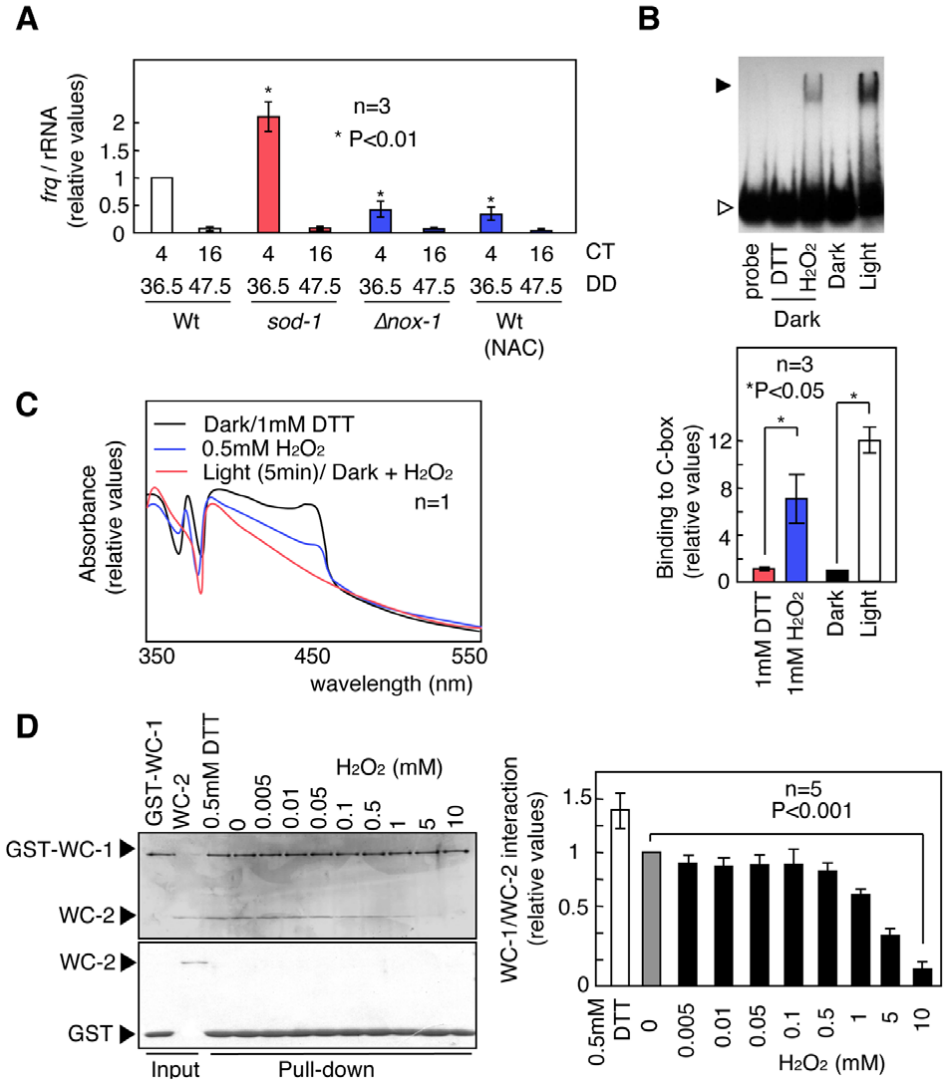
The effects of ROS on WCC function. (A) Temporal changes in *frq* mRNA expression in the race tube growth fronts from the Wt, *sod-1*, and *Δnox-1* mutants and Wt cells grown on solid medium containing the antioxidant *N*-acetyl-*L*-cysteine (NAC, 10 mM) were measured by qRT-PCR. The asterisks indicated P values compared with that at CT 4 in the Wt (B) EMSA using recombinant WC-1 and WC-2 as well as a C-box probe. We combined WCC with the probe in the dark, in the light (30 min), in the dark with 1 mM DTT and in the dark with 1 mM H_2_O_2_. The closed arrow indicates WCC bands that specifically bound to the C-box probe, and the open arrow indicates the probe. (C) Spectroscopic analysis. After treatment with DTT (1 mM) in the dark and H_2_O_2_ (0.5 mM and 1 mM) in the dark and light for 5 min, the absorbance was measured over a range of wavelength, from 350 nm to 550 nm. Light-dependent absorbance changes in recombinant WC-1 was assayed. (D) The interaction between GST-WC-1 and WC-2 under different redox conditions. The interaction between GST-WC-1 and WC-2 was assayed by a pull-down assay. The nonspecific binding between GST and WC-2 was checked as control. All values, more than three replicates, are shown as mean ± standard error (SEM).

We next addressed the way that cellular ROS regulate the circadian clock. Because light-inducible genes, such as *frq*, are transcribed by WCC, ROS may regulate light-inducible gene expression via WCC and the transcriptional function of WCC in a free-running rhythm [10], [22], [23]. To examine this possibility, we measured *frq* expression under free-running conditions. The amplitude of the rhythmic *frq* expression in *sod-1* mutants was enhanced compared with the Wt (Fig. 5A). Furthermore, the *frq* mRNA levels in a low ROS environment, such as in *Δnox-1* mutants and in cells treated with the antioxidant *N*-acetyl-*L*-cysteine (NAC) were reduced compared with those in Wt mycelia grown on untreated medium (Fig. 5A). These results suggest that cellular ROS conditions affect the expression of *frq* transcripts through WCC.

WCC binds at the *frq* promoter region, which contains a C-box and promotes *frq* expression [5], [6]. This binding is enhanced in the presence of light, both *in vitro* and *in vivo* [5]. Purified recombinant WC-1 and WC-2 proteins were tested using an electrophoretic mobility-shift assay (EMSA) to examine the effects of ROS on their binding activities. Because these recombinant proteins rapidly formed inclusion bodies after purification under normal conditions and could not be utilized for EMSAs, the purification was performed under dim red light and with the addition of the reducing reagent dithiothreitol (DTT) to prevent inactivation. DTT was removed using dialysis just before the EMSA was performed. Binding of WCC to the C-box was induced in the presence of H_2_O_2_ despite the absence of light, although the levels were 2-fold less than under illumination (Fig. 5B and Fig. S17). Flavoproteins such as VVD form flavin-cysteinyl adducts in response to light exposure and show light-dependent absorbance changes [18]. Recombinant WC-1 protein, isolated from *E. coli*, displayed light-induced absorbance changes (Fig. 5C). The light-induced absorbance of WC-1 occurred in the presence of 1 mM H_2_O_2_ despite the darkness, were reduced further in a concentration-dependent manner (Fig. 5C). Thus, ROS may promote flavin-cysteinyl adduct formation and affect the binding activity of WCC. ROS may be able to mimic the activating function of light on WC, even in the absence of light. However, the excess ROS prevented hetero-dimerization between WC-1 and WC-2 (Fig. 5D and Fig. S17). These results suggest that increased ROS (0.5–1 mM H_2_O_2_) may promote the function of WCC, but hyper-oxidative (1–10 mM H_2_O_2_) and low oxidative states (less than 0.5 mM H_2_O_2_) can inhibit its function.

## Discussion

In this study, under free-running conditions, we found two important characteristics of cellular ROS profiles. First, cellular ROS concentrations oscillate in a circadian manner (ROS oscillation). Second, the amplitude of the oscillation increases gradually with each subsequent cycle and then stabilizes (ROS homeostasis). Presumably, cellular ROS levels are maintained via a balance between the generation and the destruction of ROS. Our data indicate that most of the cellular ROS are constitutively supplied by NOX-1. With respect to ROS destruction, *Neurospora* constitutively may expresses *sod-1* transcripts that can remove excess ROS at any time. In contrast, CAT-1 and carotenoids are conditionally regulated by light, osmotic stress and circadian clocks [33], [36]. These findings suggest that SOD constitutively destroys NOX-generated ROS to a basal level, and that ROS are transiently eliminated by the rhythmic CAT-1. Furthermore, the amplitude of cellular CAT activity oscillation (including from CAT-2, CAT-3 and CCT-1/CAT-4, in addition to CAT-1) gradually decreases with each subsequent cycle. These decreases may gradually induce cellular ROS levels and maintain ROS homeostasis.

Our data suggest that cellular CAT and NOX enzymatic activity can be regulated by the circadian system. With respect to transcription levels, although *cat-1* oscillation persists in the absence of *frq*, the amplitude is stronger than in the Wt. Interestingly, the rhythmic *cat-1* expression disappears in the *Δwc-1* and *Δwc-2* mutants, unlike in the *frq^10^* mutants. A typical clock controlled gene, *ccg-1*, is regulated by FWO via an OS MAPK pathway [16]. *cat-1* is also regulated by the OS MAPK cascade [36], and FWO may control *cat-1* expression via this pathway. These results suggest that WCC may regulate the transcription of *cat-1* via the OS MAPK pathway and produce rhythmic *cat-1* expression.

The circadian system may directly or indirectly sense cellular ROS signals to control the cellular ROS levels. Genetic data suggest that increased ROS levels (as shown in *sod-1* mutants) enhance conidiation banding and rhythmic *frq* expression, whereas lower ROS levels (as in response to antioxidant treatment and in *Δnox-1* mutants) inhibit these effects (Fig. S18). Furthermore, *in vitro* analyses suggest that the transcriptional function of WCC depends on ROS levels. In these assays, H_2_O_2_ was used as a ROS and 0.1–10 mM H_2_O_2_ effectively modulated the function of WCC. Physiological H_2_O_2_ concentrations in *Neurospora* were in the range of 0.5–3.5 mM during initial growth (Table S1). These results suggest that ROS, specifically H_2_O_2_, affects the function of WCC in cells. The PAS domains in the Aer receptor function as redox sensors in *E. coli* [37]. Biochemical analyses of VVD, a PAS-LOV protein in *Neurospora*, have suggested that the oxidation of VVD causes conformational changes required for its function [38]. WC-1 and WC-2, which possess PAS or LOV domains, may also be able to sense the cellular redox state via these domains. In *Neurospora*, increased FWO amplitudes lead to a robust circadian rhythm [39]. One possible reason for the robust conidiation observed in *sod-1* mutants may be that the increased maximum ROS levels (CT 18) activate WCC and promote the accumulation of *frq* transcripts, which could lead to robust conidial banding. Meanwhile, excess ROS disassemble the hetero-complex of WC-1 and WC-2. This ROS-dependent WCC regulation may sustain ROS homeostasis. *frq*, *wc-1* and *wc-2* genetically down-regulate cellular CAT activity (Fig. 2G). The excess ROS-inhibited WCC could reduce the ROS levels via up-regulation of CATs and maintain the concentrations of ROS. As shown in the *ras-1^bd^* and *sod-1* mutants in Fig. 1D, the different ROS levels during the initial growth may stabilize to a single level via this mechanism. In general, excess ROS levels are toxic to the cell. However, redox signaling, in which ROS function as signaling molecules, has been studied in the cell from biological, physiological and pathological perspectives [40], [41]. In *Neurospora*, ROS regulate morphogenesis and conidiation banding, including mycelial and conidial development, and function as cellular signal factors independent of the circadian clock [10], [42], [43]. Therefore, a balance in cellular ROS is important for cell development.

Genetic evidence suggests that WCC up-regulates the transcription of *cat-1*, and then cellular ROS control the functions of WCC. Based on these findings, in the absence of the *frq* gene, a feedback loop composed of WCC and CAT-1 via ROS oscillation may be a candidate for a WC-FLO. During ROS oscillation, the high ROS levels may promote *cat-1* expression via WCC and reduce cellular ROS levels. Thereafter, the low ROS levels stop inducing *cat-1* via WCC, and ROS levels rise again. However, in the presence of *frq*, WCC is negatively regulated by FRQ at CT 6, despite the high levels of ROS, and the cellular H_2_O_2_ at CT 6 is 0.5 mM in Wt cells, which is sufficient to activate WCC *in vitro* (Table S1). Indeed, *cat-1^RIP^*, which lacks ROS oscillation, demonstrates normal rhythmic *frq* expression. In the presence of *frq*, maximal ROS levels would be important for these circadian behaviors in WCC.

Is ROS elimination by CAT-1 useless for the circadian system? In the mycelium, *cat-1* is induced by light and then rapidly reduces the ROS levels, counteracting the ROS generation. If ROS activates WCC, light-dependent ROS behavior as shown in Fig. 1G, may be explained because the activation of WCC should shift with photoperiodic conditions. However, the gradual decrease of light-dependent ROS levels in *cat-1^RIP^* was insufficient to influence the conidiation rhythm and light-induced *frq* expression. Meanwhile, the *sod-1* mutant, which has significantly higher ROS levels before illumination, showed high light-induced *frq* expression, and *Δnox-1*, with low ROS levels, showed lower *frq* expression than the Wt [10]. It has been proposed that VVD senses the cellular oxidative state under dark conditions and can change its conformation in response [38]. The ROS levels before irradiation may affect light-inducible *frq* expression via WCC. In any case, independent roles of *cat-1* and decreasing ROS concentrations could not be elucidated for the circadian rhythm as shown in the phenotypes observed in *cat-1^RIP^*. CAT-1 is synthesized in the growth front prior to conidial formation and may transiently reduce cellular ROS levels before conidium formation, as CAT-1 is predominantly localized to the conidium to sustain viability during germination [34]. Originally, oscillators were thought to be non-transcriptional, such as the phosphorylation cycle in cyanobacteria [44]. In *Neurospora*, the roles of *cat-1* and loss of ROS may have substituted over the course of evolution for those of *frq*, TTFL genetic oscillator.

Our data indicate that, in *Neurospora*, the cellular redox state derived from ROS and the circadian system genetically and biochemically influence each other, leading to the stabilities of cellular redox state and conidiation banding. However, we do not show data suggesting that the cellular ROS are involved in the phase and period of circadian rhythm. Cellular redox state may not specifically control the circadian clock, but this state may contribute to an environment in which TTFL can properly regulate this process. Many studies have reported that ROS are closely linked to the circadian rhythm. For instance, in humans, the cellular redox state of NAD cofactors regulates the DNA binding activity of clock complexes such as NPAS2∶BMAL1 [45]. In cyanobacteria, the circadian clock component LdpA senses the cellular redox state [46]. Recently, it was demonstrated in human red blood cells and *Osteococcus tauri* that oxidation of peroxiredoxins is involved in a TTFL-independent circadian rhythm. Genetic evidence reveals that the TTFL and the redox rhythm influence each other [47], [48]. In *Neurospora*, ROS oscillation may be a TTFL-independent circadian rhythm, but further investigation is required to improve our understanding of this process. These studies indicate that the cellular redox state can be involved in the circadian system in diverse organisms.

## Materials and Methods

### Neurospora crassa strains and growth conditions

The standard Wt strain 74-OR23-1A (FGSC #987), *ras-1^bd^* (FGSC #1859), *Δwc-1* (FGSC #11711), *Δwc-2* (FGSC #11124) and *Δnox-1* (FGSC #12867) were provided by the Fungal Genetics Stock Center at the School of Biological Sciences at the University of Missouri (Kansas City, MO, USA) [49]. The *sod-1* null, *frq^10^* single mutant, *cat-1^RIP^*, *Δwc-1*, *Δwc-2* and *Δnox-1* mutants were obtained after three successive crosses with the Wt strain in our laboratory. *cat-1^RIP^* was created using the repeat induced point-mutation (RIP) method, and CAT-1 activity was absent from *cat-1^RIP^* [34].

For conidiation banding cultures, which were used as the basis for measuring ROS, for detecting transcripts and in CAT assays, an acrylic race tube (H1 × W50 × D1 cm; the lid is a removable) was prepared, and the growing mycelial front was harvested. The clock medium (Vogel's salt mixture, 0.1% glucose, 0.17% L-arginine, 50 ng/ml biotin and 1.5% agar) was overlaid with a sterilized dialysis membrane to allow easy separation of the mycelium from the medium. The conidial suspension was inoculated onto the edge of the medium. The tube was exposed to light (20 µE m^−2^ s^−1^) for 12 hr and then kept in constant darkness. In the race tube assay, the conidia of each strain were harvested under a dim red light and inoculated onto the edge of the medium (clock medium) in a race tube [10].

### ROS measurements

The ROS assay was performed as previously described [25]. Briefly, after culturing, a 5 mm region from the *ras-1^bd^* and *sod-1* mutants, a 9 mm region from the Wt and *cat-1^RIP^* and a 7 mm region from the *frq^10^*, *Δwc-1*, *Δwc-2* and *Δnox-1* mutants were excised from the front and quickly frozen in liquid nitrogen. The size of the excised region was proportional to the growth rate of the strain. Harvesting was performed under a dim red light. The frozen samples were preserved at −80°C until analysis. Two fragments of mycelial mat were immersed in 0.5 ml of 0.2 mM lucigenin for 2 min, and the dialysis membrane was removed after vortexing for 5 sec. Luminescence, assessed as relative light units (RLUs), was measured for 20 sec in a Gene Light 55 GL-100A luminometer (MICROTEC CO., LTD, Funadashi City, Chiba, Japan). After the measurements were taken, the mycelium was dried and weighed. Luminescence values (RLU/dry weight mg) were normalized to the dry weight.

### Quantitative real-time PCR (qRT-PCR) and northern blot analysis

For qRT-PCR, the total RNA from two mycelial fragments was isolated using the RNeasy Plant Mini Kit (QIAGEN). The total RNA (1 µg) was treated with DNase I and reverse transcribed using Superscript III reverse transcriptase (Invitrogen). The primers for *frq*, *cat-1* and *ß-tub* were as follows: *frq/for* (5-cttcctgacgaccattttgtga-3), *frq/rev* (5-gtcgtccccatccaccgcttct-3), *ß-tub/for* (5-tccggcaacaagtatgtccctcgt-3), *ß-tub/rev* (5-ggcagtgaactgctcgccgat-3), *Cat-1/for* (5-gtgttgccatcatcatcgccgatggctacg-3) and *Cat-1/rev* (5-ccgcggccgcttagtacgcaatcatggag-3). All PCR reactions were carried out using SYBR® Premix EX Taq™ (Takara, Kyoto, Japan). Amplification and detection was performed on a Light Cycler 2.0 Real Time Detection System (Roche, Hercules, CA, USA). PCR was performed using the following program: 95°C (20 sec), 60°C (20 sec), and 72°C (30 sec) for 40 cycles with a final 10 sec of extension at 72°C. Fold induction values were calculated according to the equation 2^ΔΔ^Ct, indicating the differences in cycle threshold numbers between the target gene and ß-tubulin, and ΔΔCt represents the relative values in the differences between control (first harvested points and dark control in the Wt) and treatment and mutant groups. For northern blot analysis, the total RNA (20 µg) from forty mycelial fragments was separated using glyoxal-gel electrophoresis. Probes for *frq* and *cat-1* were amplified by PCR using *N. crassa* genomic DNA as a template. The primers used for the reactions were *Cat-1/for* (5-ccgaattctgtccaacatcatcagccaggc-3), *Cat-1/rev* (5- ccgcggccgcttagtacgcaatcatggag-3), *frq/for* (5-taaggaggaactgaagaggta-3) and *frq/rev* (5- gtcgtccccatccaccgcttct-3). Northern blot analysis was performed as previously described [22].

### Isolation of recombinant proteins

For the expression and purification of the recombinant WC-1 and WC-2 proteins, *wc-1* and *wc-2* cDNA were cloned using RT-PCR using the following primers: *wc1-pGEX/for* (5-ctgtcgactatgaacaacaactactacgg-3), *wc1-pGEX/rev* (5-ccgcggccgcctatacacttaagccctgttg-3), *wc2-pGEX/for* (5-ctgtcgactttgtctcacggacagcctccc-3), and *wc2-pGEX/rev* (5-aagcggccgcctatcccatatgatcgccc-3). The cDNAs were then inserted into the *Sal*I and *Not*I sites of pGEX-6P (GE Healthcare). The *E. coli* strain BL21 was transformed with either pGEX-6P-WC-1 or pGEX-6P-WC-2. WC-1 and WC-2 transformants were pre-cultured in LB medium (10% Bacto-tryptone, 5% Bacto-yeast extract and 10% NaCl) for 24 hr at 18°C and 30°C, respectively. Expression of the recombinant proteins was induced by 0.1 mM IPTG for 18 hr at 18°C for WC-1 and for 6 hr at 30°C for WC-2 in the dark. The harvested cells were resuspended in PBS buffer (10 mM Na_2_HPO_4_, 1.8 mM KH_2_PO_4_, 140 mM NaCl, 2.7 mM KCl, 1 mM DTT, and 1 mM PMSF) and lysed by sonication. Next, Triton X-100 was added to the lysate at a final concentration of 1%, and the sample was centrifuged at 15,000×g for 10 min at 4°C. Recombinant proteins were purified from the supernatant using batch purification with Glutathione Sepharose 4B (GE Healthcare). The GST tag was removed using PreScission Protease digestion for 12 hr at 4°C (GE Healthcare). Purified WC-1 was separated with a column of Sephacryl S-300HR (5×90 cm, Elution buffer: 20 mM Tris, pH 7.5, 50 mM NaCl, 1 mM DTT, GE Healthcare). The fraction of WC-1 was analyzed by SDS-PAGE. Recombinant WC-1 and WC-2 proteins were dialyzed against binding buffer (20 mM HEPES, pH 7.9, 1 mM EDTA, 2 mM MgCl_2_, 10% (v/v) glycerol and 20 µM ZnCl_2_) containing 40 mM KCl at 4°C in the dark immediately before use in the EMSA. All of these procedures were performed under a dim red light.

### Electrophoretic mobility-shift assay (EMSA)

EMSA was performed as previously described, with some modifications [5], [6]. Briefly, two oligonucleotides were used as C-box probes: *c-box/for* (5-gggcgtcctgatgccgctgcaagaccgatgacgctgcaaaattgagatcta-3) and *c-box/rev* (5-gggtagatctcaattttgcagcgtcatcggtcttgcagcggcatcaggacg-3). The oligonucleotides were annealed and end-labeled using the *Bca*BEST™ Labeling kit and ^32^P-labeled dCTP (TaKaRa). Excess dCTP in each ^32^P-labeled probe sample was removed using a Micro Bio-Spin P-30 Tris Chromatography Column (Bio-Rad), and the probe was adjusted to 18 nM. WC-1 (0.2 µg) or WC-2 (0.1 µg) was added to 1× binding buffer, 80 µM KCl, 0.1 µg poly(dI-dC), 1 µl probe, 0.5 µl rabbit serum, and 1 mM FAD in 20 µl and incubated on ice for 30 min. The reactions were loaded into 4% polyacrylamide gels containing 0.5× TBE and 2.5% (v/v) glycerol at 4°C. After electrophoresis, the radioisotopic signals were visualized using autoradiography. The binding reaction and electrophoresis were performed under a dim red light.

### Catalase activity assay

Ten mycelial fragments were disrupted in 10 mM potassium phosphate buffer (pH 7.0) by homogenization and sonication. The homogenates were centrifuged at 12,000×g for 10 min at 4°C, and the supernatant (50 µl; 50 µg protein) was added to 1 ml of reaction solution (50 mM potassium phosphate buffer, pH 7.8, and 10 mM H_2_O_2_). The decrease in absorbance (O.D. 240) after 1 min was measured using a spectrophotometer. The units are defined as 1 µmol H_2_O_2_ reduced per min per mg protein. For the in-gel assay, 20 mycelial fragments were disrupted in 10 mM potassium phosphate buffer at pH 7.0. The in-gel assay was performed as previously described [34].

### NADPH oxidase activity assay

NOX activity was evaluated as superoxide production by lucigenin-enhanced chemiluminescence [50]. Ten mycelial fragments were disrupted in 0.2 ml of extraction buffer (20 mM sodium phosphate buffer (pH 7.0), 1 mM EDTA, 1 mM PMSF, 0.5 mM leupeptin and 0.5 mM pepstatin) by sonication on ice. The homogenates (5 µl) were added to 0.5 ml of assay buffer (50 mM sodium phosphate buffer (pH 7.0), 1 mM EDTA, 150 mM sucrose and 50 µM lucigenin). After the addition of 0.1 mM NADPH, luminescence was measured as RLUs at 1-min intervals for 20 sec in a Gene Light 55 GL-100A luminometer (MICROTEC CO., LTD, Funadashi City, Chiba, Japan). NOX activity was indicated as RLUs per minute per mg of protein.

### Spectroscopic analysis

Purified recombinant WC-1 protein was exposed to light (20 µE m^−2^ s^−1^) for 3 min or treated with 0.5 or 1 mM H_2_O_2_ for 3 min in the dark. Absorbance spectra were detected using a Beckman DU 530 Spectrophotometer (Beckman Coulter, Inc.).

### Pull-down assay

Recombinant GST-WC-1 and WC-2 were purified as described above. GST-WC-1 (1.5 µg) and WC-2 (0.5 µg) were incubated in 50 µl of IP buffer (20 mM HEPES, pH 7.4, 50 mM KCl, 2 mM MgCl_2_, 0.5 mM EDTA, 0.5% NP40 and 10% glycerol) with 10 µl of Glutathione Sepharose 4B (GE Healthcare) and 1 mM DTT or 0.005 to 10 mM H_2_O_2_ at 4°C for 15 min in the dark and centrifuged at 1,000×g for 5 min at 4°C. The pellets were washed five times with 1 mL of IP buffer with 1 mM DTT or 0.005 mM H_2_O_2_ and then suspended in SDS sample buffer. The final pellets were analyzed using SDS-PAGE (8%), and stained with CBB. The binding reaction and electrophoresis were performed under a dim red light.

### Statistical analysis

All experiments were performed at least three times independently. Data are expressed as mean plus or minus standard error of the mean (SEM). Data indicating significant differences were compared using a paired Student's *t* test. Differences were considered significant when *P*<0.05.

## Supporting Information

Figure S1 Cellular ROS generation in response to growth conditions and culturing methods. (A) Wt conidia were inoculated into liquid clock medium (immersed in liquid medium) or onto sterilized dialysis membranes on solid clock medium (exposed to air) in petri dishes. The dishes were incubated at 25°C for 24 hr under constant illumination (20 µE m^−2^ s^−1^). (B) ROS generation in the mycelia immersed in liquid medium and exposed to air. The mycelia were harvested and cellular ROS levels were measured using a lucigenin chemiluminescence assay. All values are shown as mean ± standard error (SEM). (C) Conidial banding in *ras-1^bd^* mutants in an acrylic race tube (H1 × W50 × D1 cm). Scale bars indicate 1 cm. (D) Positions harvested at each CT. The growth front was harvested from the regions surrounded by the dotted lines. (E) The growth front at CT 6 and 18. Scale bars = 1 cm.
(DOC)

Figure S2 (A) Sensitivity for hydrogen peroxide in lucigenin-induced chemiluminescence. H_2_O_2_ (0.001, 0.01, 0.1, 1, 10 and 100 mM) was added to the assay solution (0.5 ml of 0.2 mM lucigenin) for 30 sec. (B) In control experiments using antioxidants, SOD (150 units/ml) or CAT-1 (30 µg/ml) was added to the assay solution containing 100 mM H_2_O_2_. Luminescence, assessed as relative light units (RLUs), was measured for 20 sec in a Gene Light 55 GL-100A luminometer (MICROTEC CO., LTD, Funadashi City, Chiba, Japan). These results indicate that lucigenin can detect H_2_O_2_ more than 0.1 mM.
(DOC)

Figure S3 Cellular ROS generation in the growth front at CT 6 and 18. The growth front at CT 6 includes conidia, and the conidial dry weight and carotenoids may influence cellular ROS levels. Therefore only mycelia (1 mm width from front) in the growth front were harvested at CT 6 and 18. Cellular ROS levels were then measured using the lucigenin chemiluminescence assay. ROS oscillation in the mycelium of the growth front was also detected. All values are shown as mean ± standard error (SEM).
(DOC)

Figure S4 Detection of cellular ROS in the mycelium using NBT and Diogenes. (A) NBT staining in the growth front corresponding to circadian times (CT) 6 and 18 in *ras-1^bd^* mutants. Mycelia turn blue when NBT is reduced by O_2_^−^. (B) Cellular ROS detection using Diogenes reagents in *ras-1^bd^* mutants. All values are shown as mean ± standard error (SEM) (see Methods S1).
(DOC)

Figure S5 Transient cellular H_2_O_2_ levels. (A) Cellular H_2_O_2_ levels under constant darkness in Wt cells. Cellular H_2_O_2_ levels in race tube growth fronts were measured using the H_2_O_2_ assay described above. Relative values were calculated using the values obtained at CT 6. (B) Cellular H_2_O_2_ levels in the Wt and *sod-1* mutants at CT 6 and CT 18. Cellular H_2_O_2_ levels in race tube growth fronts were measured. Relative values were calculated using the values obtained for the Wt at CT 6. Cellular H_2_O_2_ levels in Wt cells displayed small oscillations and were slightly lower than in the *sod-1* mutants. All values are shown as mean ± standard error (SEM) (see Methods S1).
(DOC)

Figure S6 Effects of entrainment on cellular ROS. Wt race tube mycelia were exposed to light at CT 6 and 18, and the temperature was shifted from 25°C to 35°C or 15°C for 1 hr. ROS levels were measured using the lucigenin chemiluminescence assay. All values are shown as mean ± standard error (SEM).
(DOC)

Figure S7 The effect of light on total CAT activity. Mycelia (Wt) in the race tube growth front at CT 18 were exposed to light for 1 hr. The total CAT activity was determined using a spectrometric assay. All values are shown as mean ± standard error (SEM).
(DOC)

Figure S8 The effect of the CAT inhibitor 3-AT on conidiation banding. (A) Total CAT activity in mycelia (Wt) grown in the medium containing 3-AT. Mycelia in the race tube growth front at CT 6 (DD 16.5 hr) were harvested, and the total CAT activity was determined using a spectrometric assay. (B) Cellular ROS and H_2_O_2_ levels in mycelia grown on medium containing 3-AT. Mycelia in the race tube growth front were harvested at CT 18 (DD 16.5 hr). Cellular ROS and H_2_O_2_ levels were determined using the lucigenin chemiluminescence assay and a hydrogen peroxide assay, respectively. To determine H_2_O_2_ levels, relative values were calculated based on the values obtained for the control. Treatment with 3-AT inhibited total CAT activity and caused an increase in cellular ROS and H_2_O_2_ levels. (C) Conidiation banding in Wt cells on medium containing 3-AT. All values are shown as mean ± standard error (SEM).
(DOC)

Figure S9 Cellular ROS generation at CT 18 in subsequent cycles in Wt and *cat-1^RIP^* cells. Mycelia in the race tube growth front were harvested at CT 18 of every cycle (DD 27.5, 49.5 and 71.5 hr), and cellular ROS levels were determined using the lucigenin chemiluminescence assay. All values are shown as mean ± standard error (SEM).
(DOC)

Figure S10 Conidiation banding in a *cat-1* loss-of-function mutant. The race tubes were exposed to light (light-resetting) for 12 hr after inoculation and then transferred to constant darkness (DD). The growth front was marked at 24-hr intervals.
(DOC)

Figure S11 Conidiation banding in a *cat-1* loss-of-function mutant under a 12-h light/12-h dark cycle. The growth front was marked at 24-h intervals (n = 12).
(DOC)

Figure S12 Clock-controlled and light-induced *frq* expression. (A) *frq* mRNA accumulation in race tube growth fronts of Wt and *cat-1^RIP^* cells at CT 4 and 16. *frq* transcripts were detected using RT-PCR with *ß-tub* as the quantitation control. (B) Light-induced *frq* mRNA accumulation in the Wt and *cat-1^RIP^*. Mycelia in race tube growth fronts were exposed to light for 15 min (L15) and 60 min (L60) at CT 24/0 (DD 11 h). *frq* transcripts were detected using RT-PCR with *ß-tub* as the quantitation control. All values are shown as mean ± standard error (SEM) (see Methods S1).
(DOC)

Figure S13 Control NOX activity assay. Mycelia harvested at CT 18 were disrupted and used as cell lysates in the assay. The antioxidants SOD (150 units/ml) or CAT-1 (30 µg/ml) were added to the assay buffer. Cell lysates and NADPH were added to the assay buffer at the points noted using closed and open arrows, respectively. Temporal ROS generation (RLU/mg protein) by NOX was detected using lucigenin chemiluminescence. This experiment indicates that ROS generated in this assay are superoxide radicals, but not H_2_O_2_ and that NADPH-dependent superoxide generation is due to NOX activity (see Methods S1).
(DOC)

Figure S14 Conidiation banding in *Δnox-1* under constant darkness after 12 hr of light. The growth front was marked at 24-hr intervals (n = 12).
(DOC)

Figure S15 Conidiation banding in *Δnox-1* under a 12-hr light/12-hr dark cycle. The growth front was marked at 24-hr intervals (n = 12).
(DOC)

Figure S16 Profiles of cellular NOX activity in Wt cells. (A) Temporal cellular NOX activity. Mycelia in the race tube growth front were harvested at CT 0, 6, 12, 18 and 24. Cellular NOX activity was measured and calculated as RLU/min/mg protein. The values shown are as relative values based on the values obtained at 11 DD. (B) Cellular NOX activity at CT 18 in subsequent cycles in Wt cells. Mycelia in the race tube growth front were harvested at CT 18 of every cycle (DD 27.5–115.5 h). Cellular NOX activities were measured and calculated as RLU/min/mg protein. The values shown are as relative values based on the values obtained at 27.5 DD. All values are shown as mean ± standard error (SEM).
(DOC)

Figure S17 Control EMSA and pull-down assay. (A) EMSA control experiment using antioxidants. SOD (1,500 units/ml) or CAT-1 (75 µg/ml) was added to the reaction mixture containing 1 mM H_2_O_2_. (B) Control pull-down assay using antioxidants. SOD (1,500 units/ml) or CAT-1 (75 µg/ml) was added to the reaction mixture containing 10 mM H_2_O_2_ (see Methods S1).
(DOC)

Figure S18 Correlations between cellular ROS levels, *frq* expression and conidiation banding under free-running conditions in the Wt, *cat-1^RIP^*, *sod-1* and *Δnox-1*. In the graph showing cellular ROS levels and *frq* expression, red lines indicate the levels in each strain and dotted lines indicate the Wt.
(DOC)

Methods S1 Materials and methods for supporting data.
(DOC)

Table S1 H_2_O_2_ concentration in Wt mycelial cells. Cellular volumes per 15,000 µm^2^ in mycelial fragments were determined from the microscopic images. Based on these values, the total volumes in a mycelial fragment (9×10 mm size) were calculated. H_2_O_2_ concentration in one fragment of mycelial mats was measured by BIOXYTECH Hydrogen Peroxide Assay kit. The cellular H_2_O_2_ concentrations calculated from the cellular volumes and H_2_O_2_ amounts.
(DOC)
