# Supplementary material for: Cross-Talk between the Cellular Redox State and the Circadian System in Neurospora
Source: PLoS One. 2011 Dec 2;6(12):e28227. doi: 10.1371/journal.pone.0028227 (PMC3229512; doi:10.1371/journal.pone.0028227)
Supplement: Figure S1 — Cellular ROS generation in response to growth conditions and culturing methods. (A) Wt conidia were inoculated into liquid clock medium (immersed in liquid medium) or onto sterilized dialysis membranes on solid clock medium (exposed to air) in petri dishes. The dishes were incubated at 25°C for 24 hr under constant illumination (20 µE m−2 s−1). (B) ROS generation in the mycelia immersed in liquid medium and exposed to air. The mycelia were harvested and cellular ROS levels were measured using a lucigenin chemiluminescence assay. All values are shown as mean ± standard error (SEM). (C) Conidial banding in ras-1bd mutants in an acrylic race tube (H1 × W50 × D1 cm). Scale bars indicate 1 cm. (D) Positions harvested at each CT. The growth front was harvested from the regions surrounded by the dotted lines. (E) The growth front at CT 6 and 18. Scale bars = 1 cm. (DOC) [file pone.0028227.s001.doc]

**
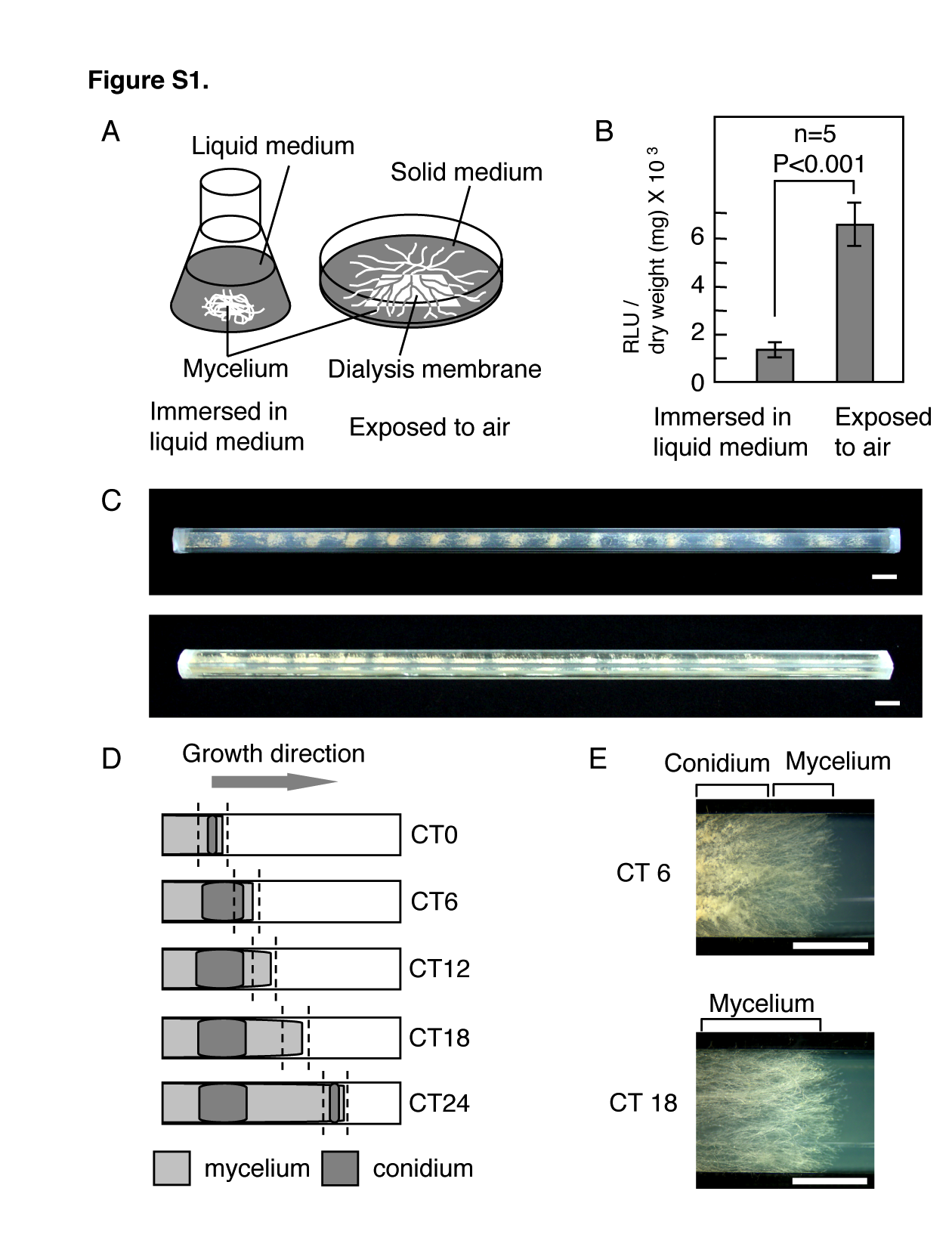
**

**Figure S1.** Cellular ROS generation in response to growth conditions and culturing methods. (A) Wt conidia were inoculated into liquid clock medium (immersed in liquid medium) or onto sterilized dialysis membranes on solid clock medium (exposed to air) in petri dishes. The dishes were incubated at 25 ˚C for 24 hr under constant illumination (20 µE m^-2^ s^-1^). (B) ROS generation in the mycelia immersed in liquid medium and exposed to air. The mycelia were harvested and cellular ROS levels were measured using a lucigenin chemiluminescence assay. All values are shown as mean ± standard error (SEM). (C) Conidial banding in *ras-1^bd^* mutants in an acrylic race tube (H1 x W50 x D1 cm). Scale bars indicate 1 cm. (D) Positions harvested at each CT. The growth front was harvested from the regions surrounded by the dotted lines. (E) The growth front at CT 6 and 18. Scale bars = 1 cm.
