# Supplementary material for: Cross-Talk between the Cellular Redox State and the Circadian System in Neurospora
Source: PLoS One. 2011 Dec 2;6(12):e28227. doi: 10.1371/journal.pone.0028227 (PMC3229512; doi:10.1371/journal.pone.0028227)
Supplement: Figure S2 — (A) Sensitivity for hydrogen peroxide in lucigenin-induced chemiluminescence. H2O2 (0.001, 0.01, 0.1, 1, 10 and 100 mM) was added to the assay solution (0.5 ml of 0.2 mM lucigenin) for 30 sec. (B) In control experiments using antioxidants, SOD (150 units/ml) or CAT-1 (30 µg/ml) was added to the assay solution containing 100 mM H2O2. Luminescence, assessed as relative light units (RLUs), was measured for 20 sec in a Gene Light 55 GL-100A luminometer (MICROTEC CO., LTD, Funadashi City, Chiba, Japan). These results indicate that lucigenin can detect H2O2 more than 0.1 mM. (DOC) [file pone.0028227.s002.doc]

**
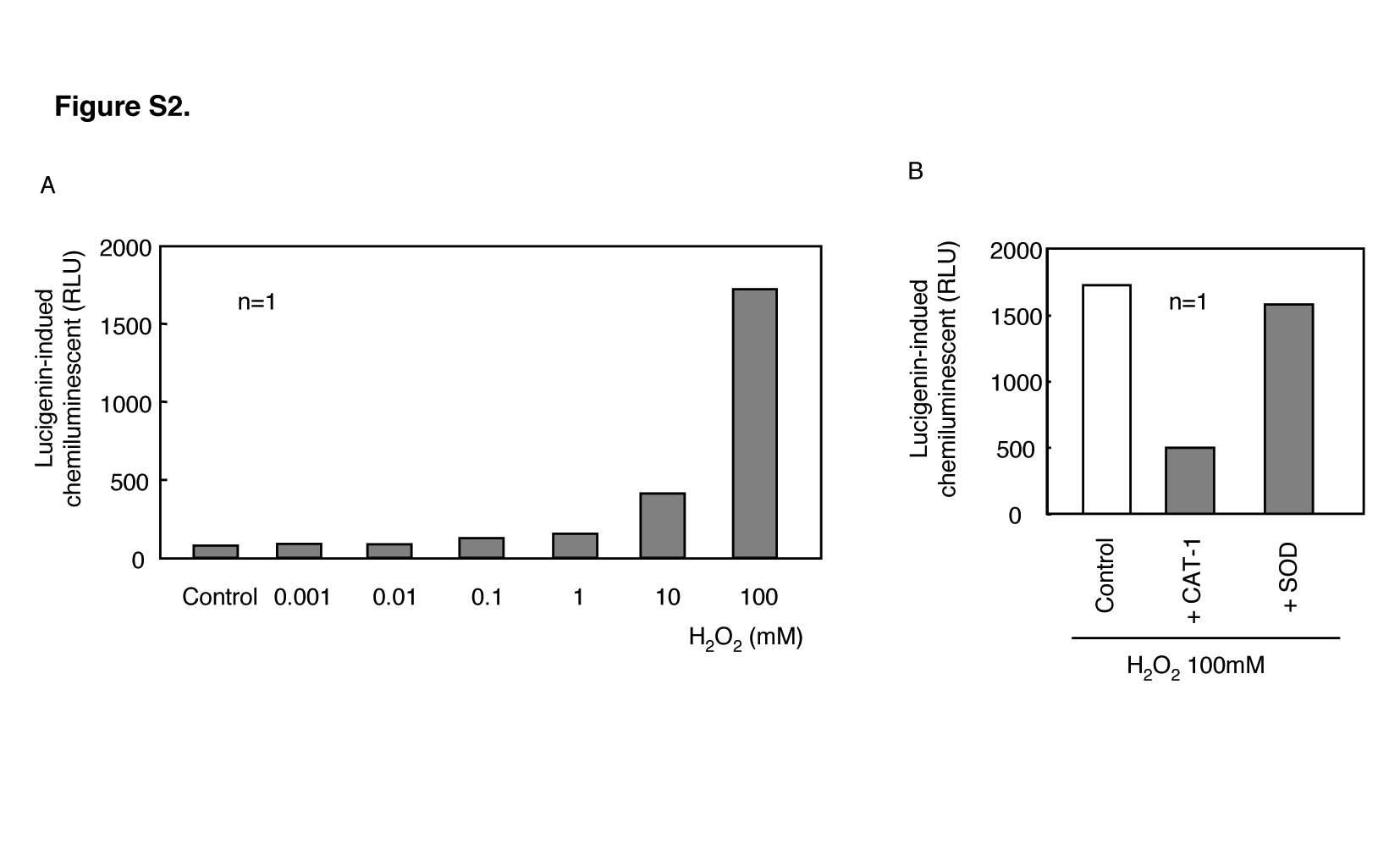
**

**Figure S2.** (A) Sensitivity for hydrogen peroxide in lucigenin-induced chemiluminescence. H_2_O_2_ (0.001, 0.01, 0.1, 1, 10 and 100 mM) was added to the assay solution (0.5 ml of 0.2 mM lucigenin) for 30 sec. (B) In control experiments using antioxidants, SOD (150 units/ml) or CAT-1 (30 µg/ml) was added to the assay solution containing 100 mM H_2_O_2_. Luminescence, assessed as relative light units (RLUs), was measured for 20 sec in a Gene Light 55 GL-100A luminometer (MICROTEC CO., LTD, Funadashi City, Chiba, Japan). These results indicate that lucigenin can detect H_2_O_2_ more than 0.1 mM.
