# Supplementary material for: Cross-Talk between the Cellular Redox State and the Circadian System in Neurospora
Source: PLoS One. 2011 Dec 2;6(12):e28227. doi: 10.1371/journal.pone.0028227 (PMC3229512; doi:10.1371/journal.pone.0028227)
Supplement: Figure S3 — Cellular ROS generation in the growth front at CT 6 and 18. The growth front at CT 6 includes conidia, and the conidial dry weight and carotenoids may influence cellular ROS levels. Therefore only mycelia (1 mm width from front) in the growth front were harvested at CT 6 and 18. Cellular ROS levels were then measured using the lucigenin chemiluminescence assay. ROS oscillation in the mycelium of the growth front was also detected. All values are shown as mean ± standard error (SEM). (DOC) [file pone.0028227.s003.doc]

**
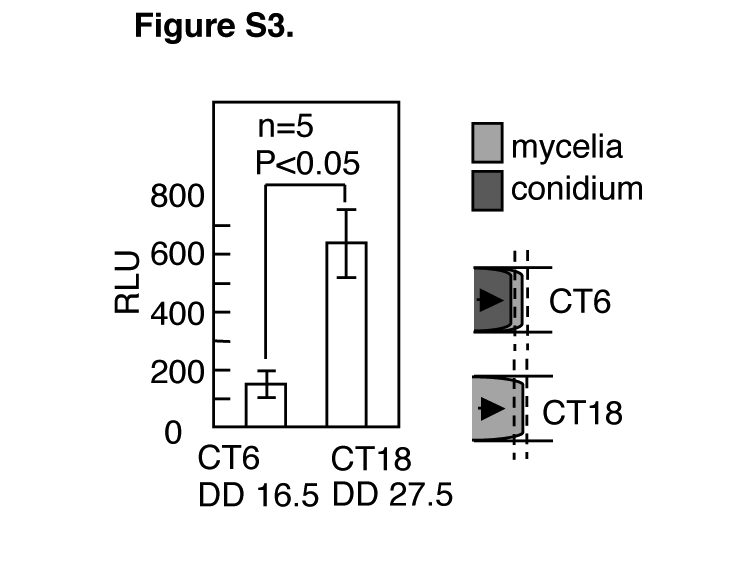
**

**Figure S3.** Cellular ROS generation in the growth front at CT 6 and 18. The growth front at CT 6 includes conidia, and the conidial dry weight and carotenoids may influence cellular ROS levels. Therefore only mycelia (1 mm width from front) in the growth front were harvested at CT 6 and 18. Cellular ROS levels were then measured using the lucigenin chemiluminescence assay. ROS oscillation in the mycelium of the growth front was also detected. All values are shown as mean ± standard error (SEM).
