# Supplementary material for: Cross-Talk between the Cellular Redox State and the Circadian System in Neurospora
Source: PLoS One. 2011 Dec 2;6(12):e28227. doi: 10.1371/journal.pone.0028227 (PMC3229512; doi:10.1371/journal.pone.0028227)
Supplement: Figure S4 — Detection of cellular ROS in the mycelium using NBT and Diogenes. (A) NBT staining in the growth front corresponding to circadian times (CT) 6 and 18 in ras-1bd mutants. Mycelia turn blue when NBT is reduced by O2 −. (B) Cellular ROS detection using Diogenes reagents in ras-1bd mutants. All values are shown as mean ± standard error (SEM) (see Methods S1). (DOC) [file pone.0028227.s004.doc]

**
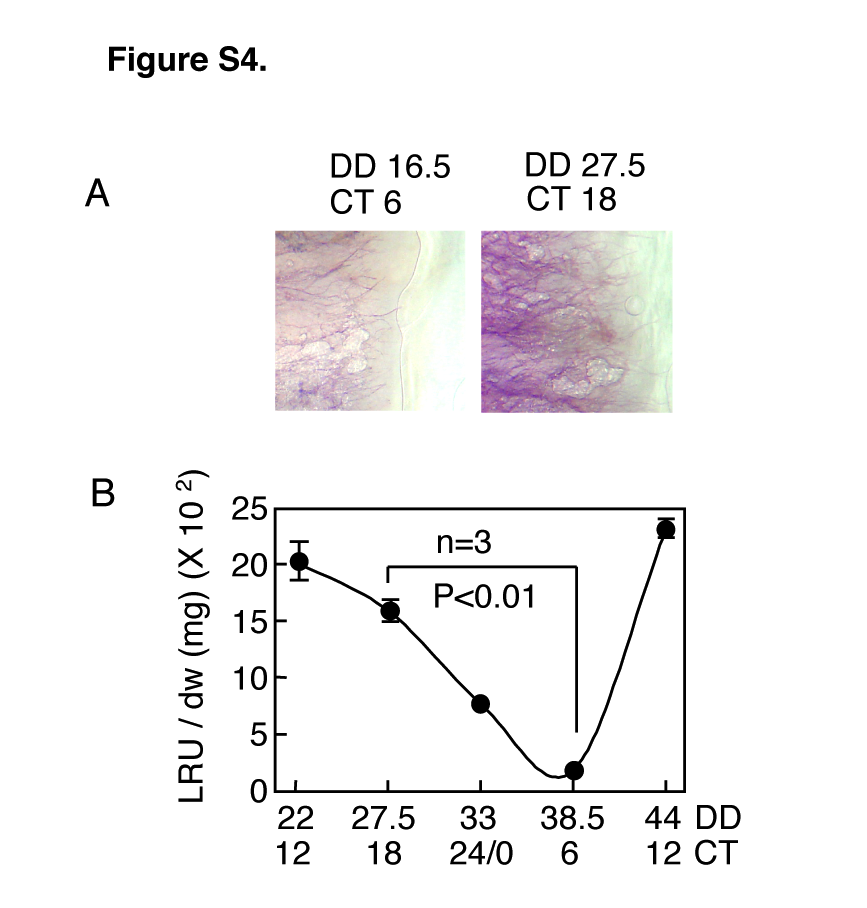
**

**Figure S4.** Detection of cellular ROS in the mycelium using NBT and Diogenes. (A**)** NBT staining in the growth front corresponding to circadian times (CT) 6 and 18 in *ras-1^bd^* mutants. Mycelia turn blue when NBT is reduced by O_2_^.-^. (B**)** Cellular ROS detection using Diogenes reagents in *ras-1^bd^* mutants. All values are shown as mean ± standard error (SEM) (see Methods S1).
