# Supplementary material for: Cross-Talk between the Cellular Redox State and the Circadian System in Neurospora
Source: PLoS One. 2011 Dec 2;6(12):e28227. doi: 10.1371/journal.pone.0028227 (PMC3229512; doi:10.1371/journal.pone.0028227)
Supplement: Figure S5 — Transient cellular H2O2 levels. (A) Cellular H2O2 levels under constant darkness in Wt cells. Cellular H2O2 levels in race tube growth fronts were measured using the H2O2 assay described above. Relative values were calculated using the values obtained at CT 6. (B) Cellular H2O2 levels in the Wt and sod-1 mutants at CT 6 and CT 18. Cellular H2O2 levels in race tube growth fronts were measured. Relative values were calculated using the values obtained for the Wt at CT 6. Cellular H2O2 levels in Wt cells displayed small oscillations and were slightly lower than in the sod-1 mutants. All values are shown as mean ± standard error (SEM) (see Methods S1). (DOC) [file pone.0028227.s005.doc]

**
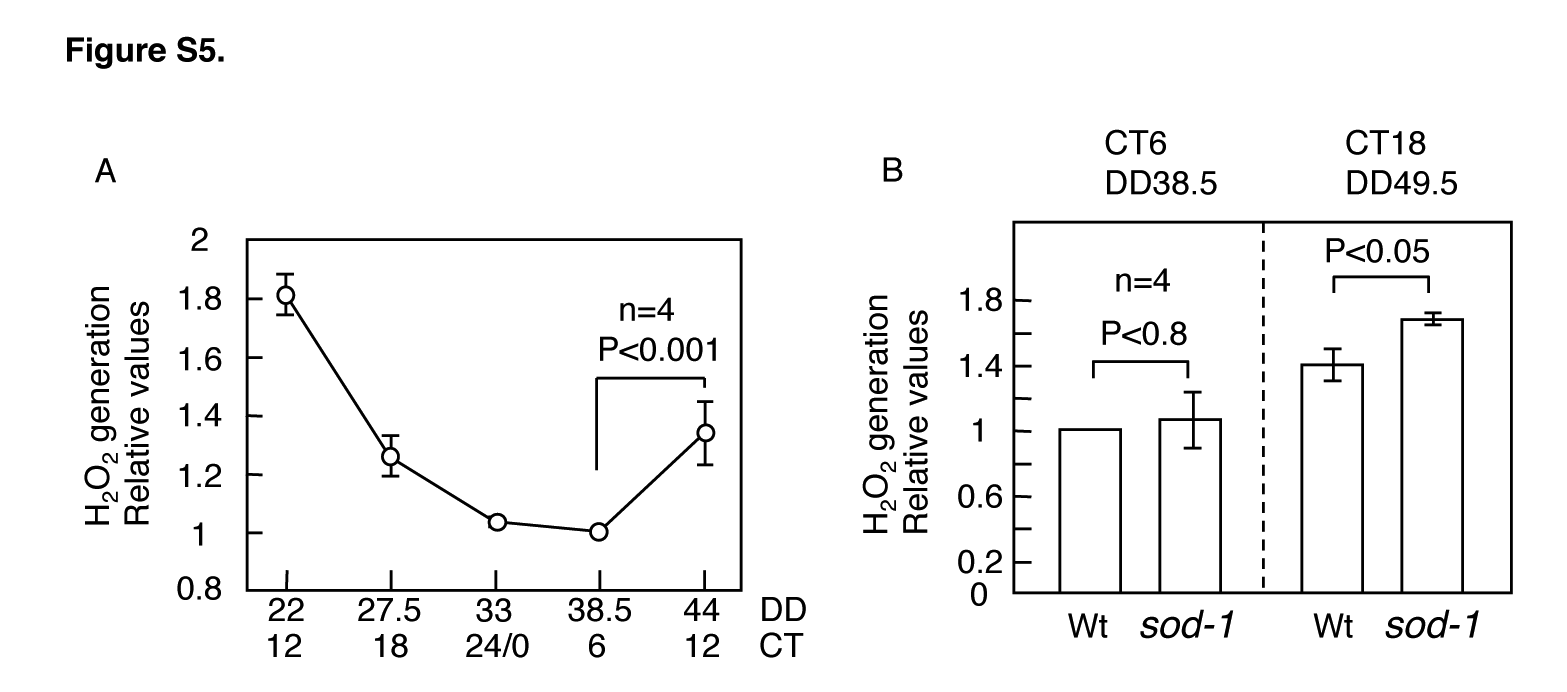
**

**Figure S5.** Transient cellular H_2_O_2_ levels. (A) Cellular H_2_O_2_ levels under constant darkness in Wt cells. Cellular H_2_O_2_ levels in race tube growth fronts were measured using the H_2_O_2_ assay described above. Relative values were calculated using the values obtained at CT 6. (B) Cellular H_2_O_2_ levels in the Wt and *sod-1* mutants at CT 6 and CT 18. Cellular H_2_O_2_ levels in race tube growth fronts were measured. Relative values were calculated using the values obtained for the Wt at CT 6. Cellular H_2_O_2_ levels in Wt cells displayed small oscillations and were slightly lower than in the *sod-1* mutants. All values are shown as mean ± standard error (SEM) (see Methods S1).
