# Supplementary material for: Cross-Talk between the Cellular Redox State and the Circadian System in Neurospora
Source: PLoS One. 2011 Dec 2;6(12):e28227. doi: 10.1371/journal.pone.0028227 (PMC3229512; doi:10.1371/journal.pone.0028227)
Supplement: Figure S6 — Effects of entrainment on cellular ROS. Wt race tube mycelia were exposed to light at CT 6 and 18, and the temperature was shifted from 25°C to 35°C or 15°C for 1 hr. ROS levels were measured using the lucigenin chemiluminescence assay. All values are shown as mean ± standard error (SEM). (DOC) [file pone.0028227.s006.doc]

**
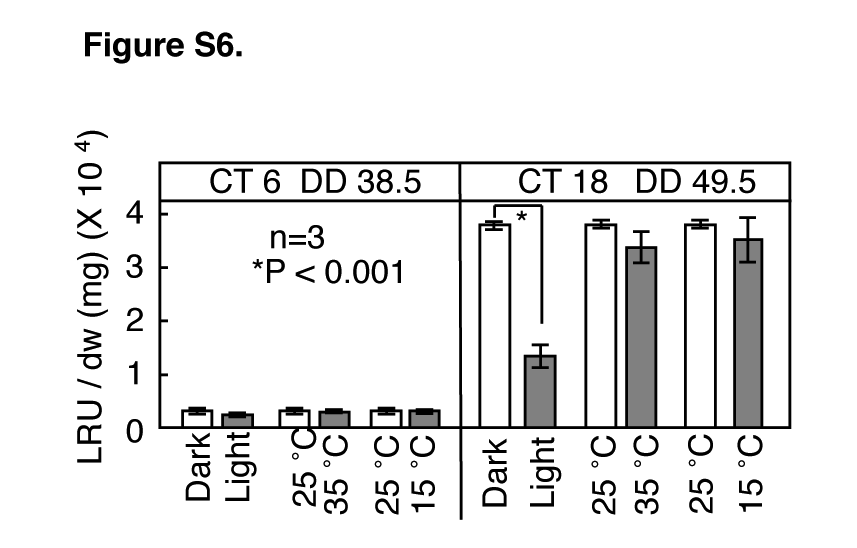
**

**Figure S6.** Effects of entrainment on cellular ROS. Wt race tube mycelia were exposed to light at CT 6 and 18, and the temperature was shifted from 25 ˚C to 35 ˚C or 15 ˚C for 1 hr. ROS levels were measured using the lucigenin chemiluminescence assay. All values are shown as mean ± standard error (SEM).
