# Supplementary material for: Cross-Talk between the Cellular Redox State and the Circadian System in Neurospora
Source: PLoS One. 2011 Dec 2;6(12):e28227. doi: 10.1371/journal.pone.0028227 (PMC3229512; doi:10.1371/journal.pone.0028227)
Supplement: Figure S7 — The effect of light on total CAT activity. Mycelia (Wt) in the race tube growth front at CT 18 were exposed to light for 1 hr. The total CAT activity was determined using a spectrometric assay. All values are shown as mean ± standard error (SEM). (DOC) [file pone.0028227.s007.doc]

**
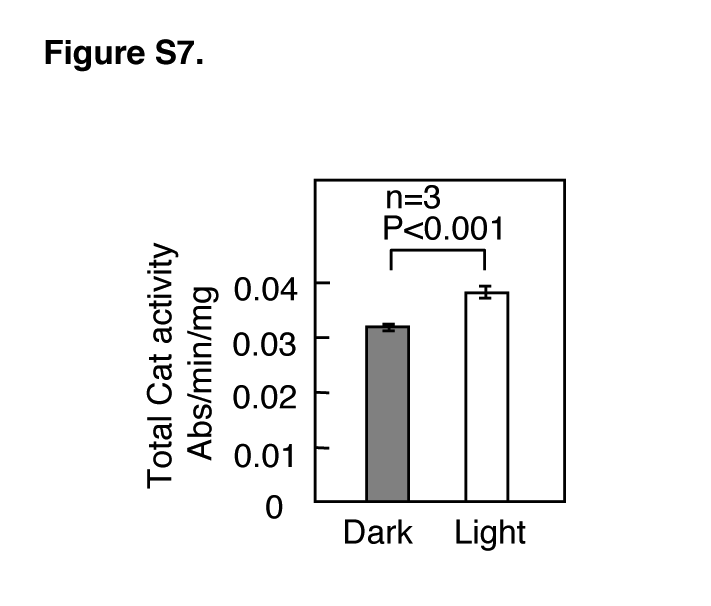
**

**Figure S7.** The effect of light on total CAT activity. Mycelia (Wt) in the race tube growth front at CT 18 were exposed to light for 1 hr. The total CAT activity was determined using a spectrometric assay. All values are shown as mean ± standard error (SEM).
