# Supplementary material for: Cross-Talk between the Cellular Redox State and the Circadian System in Neurospora
Source: PLoS One. 2011 Dec 2;6(12):e28227. doi: 10.1371/journal.pone.0028227 (PMC3229512; doi:10.1371/journal.pone.0028227)
Supplement: Figure S8 — The effect of the CAT inhibitor 3-AT on conidiation banding. (A) Total CAT activity in mycelia (Wt) grown in the medium containing 3-AT. Mycelia in the race tube growth front at CT 6 (DD 16.5 hr) were harvested, and the total CAT activity was determined using a spectrometric assay. (B) Cellular ROS and H2O2 levels in mycelia grown on medium containing 3-AT. Mycelia in the race tube growth front were harvested at CT 18 (DD 16.5 hr). Cellular ROS and H2O2 levels were determined using the lucigenin chemiluminescence assay and a hydrogen peroxide assay, respectively. To determine H2O2 levels, relative values were calculated based on the values obtained for the control. Treatment with 3-AT inhibited total CAT activity and caused an increase in cellular ROS and H2O2 levels. (C) Conidiation banding in Wt cells on medium containing 3-AT. All values are shown as mean ± standard error (SEM). (DOC) [file pone.0028227.s008.doc]

**
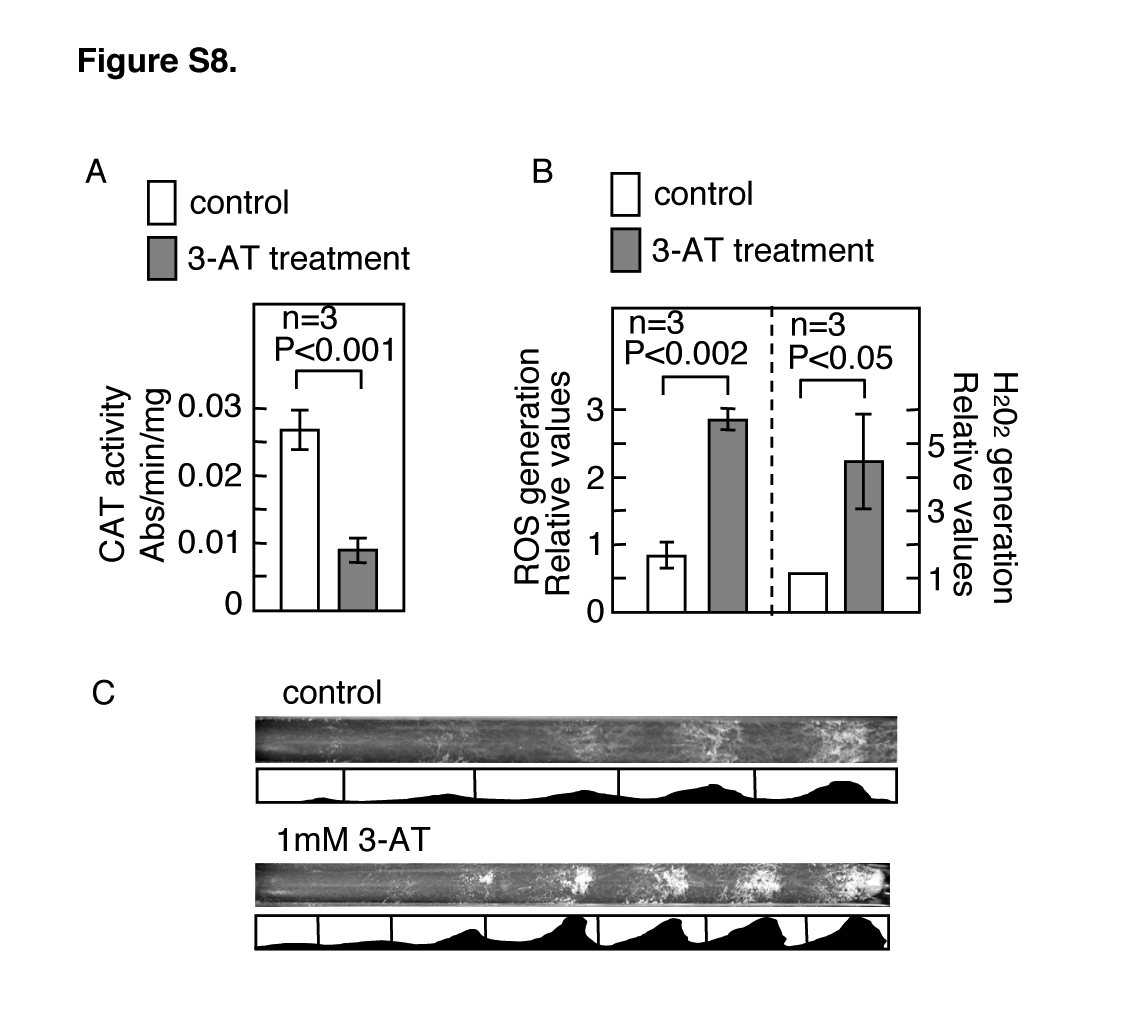
**

**Figure S8.** The effect of the CAT inhibitor 3-AT on conidiation banding. (A) Total CAT activity in mycelia (Wt) grown in the medium containing 3-AT. Mycelia in the race tube growth front at CT 6 (DD 16.5 hr) were harvested, and the total CAT activity was determined using a spectrometric assay. (B) Cellular ROS and H_2_O_2_ levels in mycelia grown on medium containing 3-AT. Mycelia in the race tube growth front were harvested at CT 18 (DD 16.5 hr). Cellular ROS and H_2_O_2_ levels were determined using the lucigenin chemiluminescence assay and a hydrogen peroxide assay, respectively. To determine H_2_O_2_ levels, relative values were calculated based on the values obtained for the control. Treatment with 3-AT inhibited total CAT activity and caused an increase in cellular ROS and H_2_O_2_ levels. (C) Conidiation banding in Wt cells on medium containing 3-AT. All values are shown as mean ± standard error (SEM).
