# Supplementary material for: Cross-Talk between the Cellular Redox State and the Circadian System in Neurospora
Source: PLoS One. 2011 Dec 2;6(12):e28227. doi: 10.1371/journal.pone.0028227 (PMC3229512; doi:10.1371/journal.pone.0028227)
Supplement: Figure S9 — Cellular ROS generation at CT 18 in subsequent cycles in Wt and cat-1RIP cells. Mycelia in the race tube growth front were harvested at CT 18 of every cycle (DD 27.5, 49.5 and 71.5 hr), and cellular ROS levels were determined using the lucigenin chemiluminescence assay. All values are shown as mean ± standard error (SEM). (DOC) [file pone.0028227.s009.doc]

**
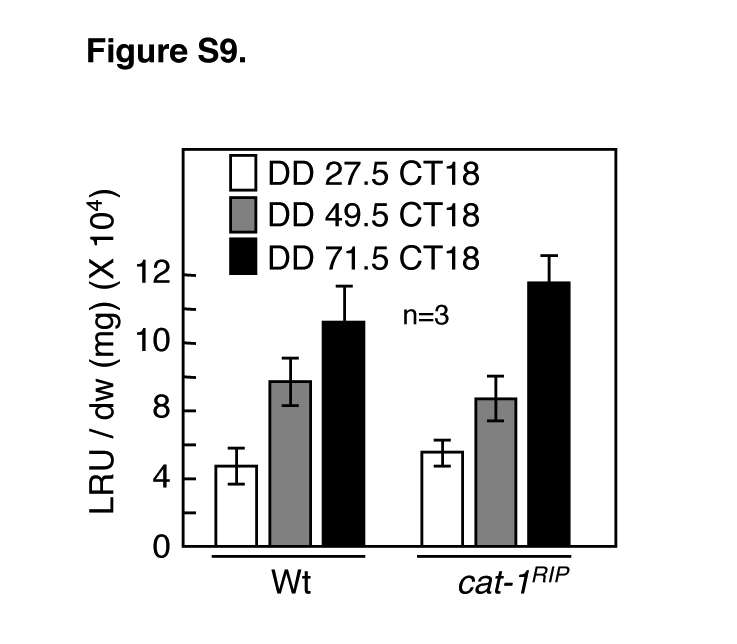
**

**Figure S9.** Cellular ROS generation at CT 18 in subsequent cycles in Wt and *cat-1^RIP^* cells. Mycelia in the race tube growth front were harvested at CT 18 of every cycle (DD 27.5, 49.5 and 71.5 hr), and cellular ROS levels were determined using the lucigenin chemiluminescence assay. All values are shown as mean ± standard error (SEM).
