# Supplementary material for: Cross-Talk between the Cellular Redox State and the Circadian System in Neurospora
Source: PLoS One. 2011 Dec 2;6(12):e28227. doi: 10.1371/journal.pone.0028227 (PMC3229512; doi:10.1371/journal.pone.0028227)
Supplement: Figure S10 — Conidiation banding in a cat-1 loss-of-function mutant. The race tubes were exposed to light (light-resetting) for 12 hr after inoculation and then transferred to constant darkness (DD). The growth front was marked at 24-hr intervals. (DOC) [file pone.0028227.s010.doc]

**
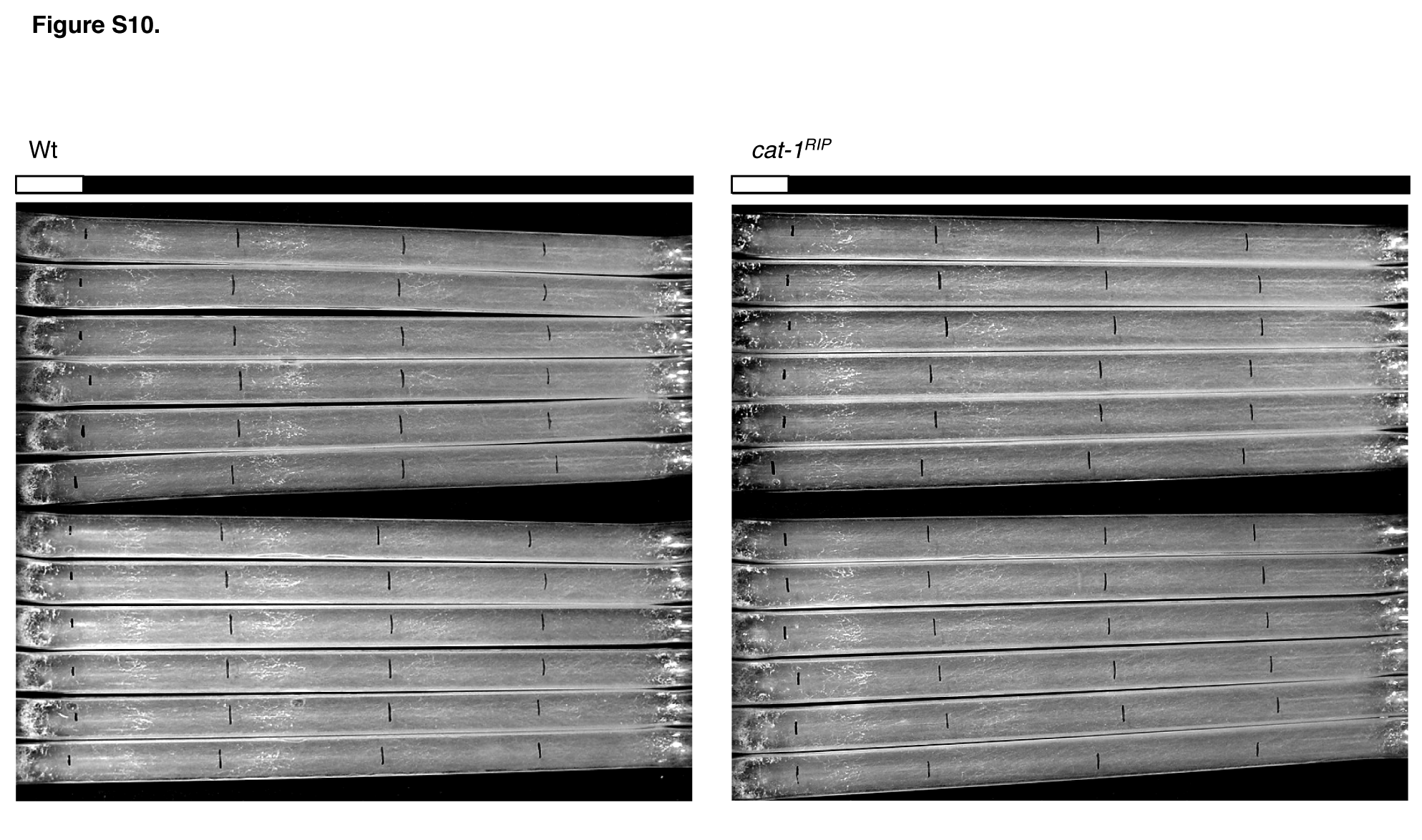
**

**Figure S10.** Conidiation banding in a *cat-1* loss-of-function mutant. The race tubes were exposed to light (light-resetting) for 12 hr after inoculation and then transferred to constant darkness (DD). The growth front was marked at 24-hr intervals.
