# Supplementary material for: Cross-Talk between the Cellular Redox State and the Circadian System in Neurospora
Source: PLoS One. 2011 Dec 2;6(12):e28227. doi: 10.1371/journal.pone.0028227 (PMC3229512; doi:10.1371/journal.pone.0028227)
Supplement: Figure S11 — Conidiation banding in a cat-1 loss-of-function mutant under a 12-h light/12-h dark cycle. The growth front was marked at 24-h intervals (n = 12). (DOC) [file pone.0028227.s011.doc]

**
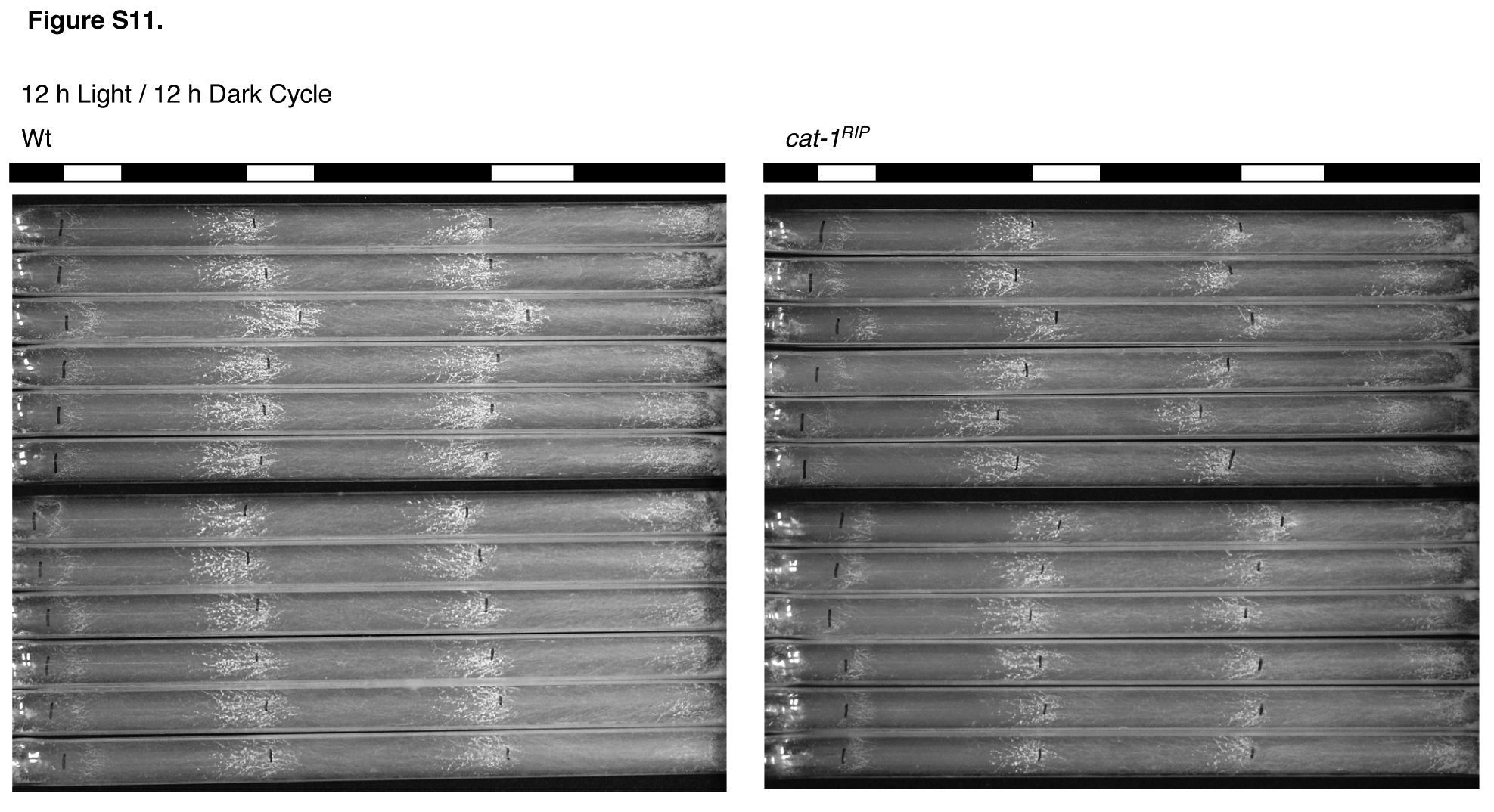
**

**Figure S11.** Conidiation banding in a *cat-1* loss-of-function mutant under a 12-h light/12-h dark cycle. The growth front was marked at 24-h intervals (n = 12).
