# Supplementary material for: Cross-Talk between the Cellular Redox State and the Circadian System in Neurospora
Source: PLoS One. 2011 Dec 2;6(12):e28227. doi: 10.1371/journal.pone.0028227 (PMC3229512; doi:10.1371/journal.pone.0028227)
Supplement: Figure S12 — Clock-controlled and light-induced frq expression. (A) frq mRNA accumulation in race tube growth fronts of Wt and cat-1RIP cells at CT 4 and 16. frq transcripts were detected using RT-PCR with ß-tub as the quantitation control. (B) Light-induced frq mRNA accumulation in the Wt and cat-1RIP. Mycelia in race tube growth fronts were exposed to light for 15 min (L15) and 60 min (L60) at CT 24/0 (DD 11 h). frq transcripts were detected using RT-PCR with ß-tub as the quantitation control. All values are shown as mean ± standard error (SEM) (see Methods S1). (DOC) [file pone.0028227.s012.doc]

**
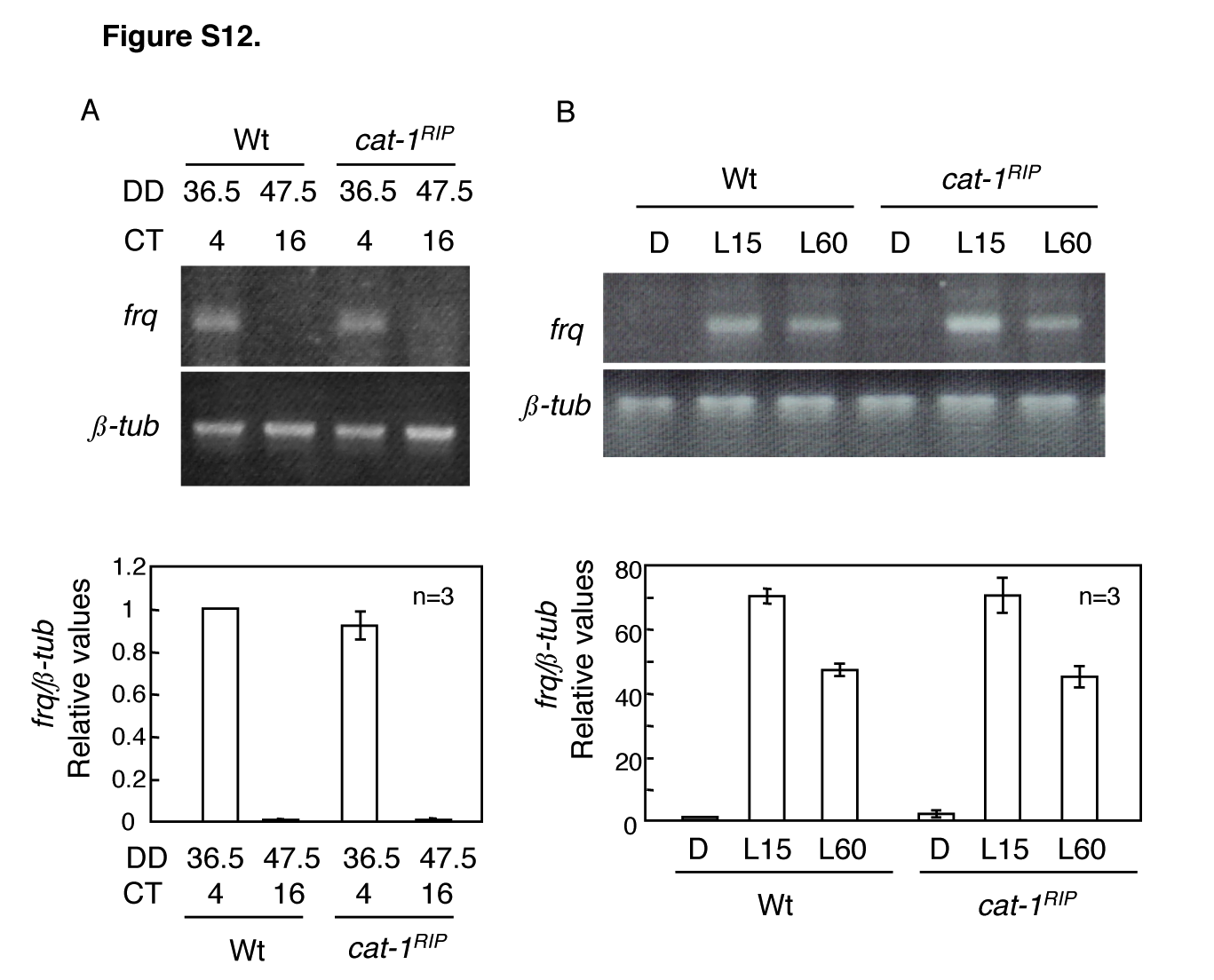
**

**Figure S12.** Clock-controlled and light-induced *frq* expression. (A) *frq* mRNA accumulation in race tube growth fronts of Wt and *cat-1^RIP^* cells at CT 4 and 16. *frq* transcripts were detected using RT-PCR with *ß-tub* as the quantitation control. (B) Light-induced *frq* mRNA accumulation in the Wt and *cat-1^RIP^.* Mycelia in race tube growth fronts were exposed to light for 15 min (L15) and 60 min (L60) at CT 24/0 (DD 11 h). *frq* transcripts were detected using RT-PCR with *ß-tub* as the quantitation control. All values are shown as mean ± standard error (SEM) (see Methods S1).
