# Supplementary material for: Cross-Talk between the Cellular Redox State and the Circadian System in Neurospora
Source: PLoS One. 2011 Dec 2;6(12):e28227. doi: 10.1371/journal.pone.0028227 (PMC3229512; doi:10.1371/journal.pone.0028227)
Supplement: Figure S13 — Control NOX activity assay. Mycelia harvested at CT 18 were disrupted and used as cell lysates in the assay. The antioxidants SOD (150 units/ml) or CAT-1 (30 µg/ml) were added to the assay buffer. Cell lysates and NADPH were added to the assay buffer at the points noted using closed and open arrows, respectively. Temporal ROS generation (RLU/mg protein) by NOX was detected using lucigenin chemiluminescence. This experiment indicates that ROS generated in this assay are superoxide radicals, but not H2O2 and that NADPH-dependent superoxide generation is due to NOX activity (see Methods S1). (DOC) [file pone.0028227.s013.doc]

**
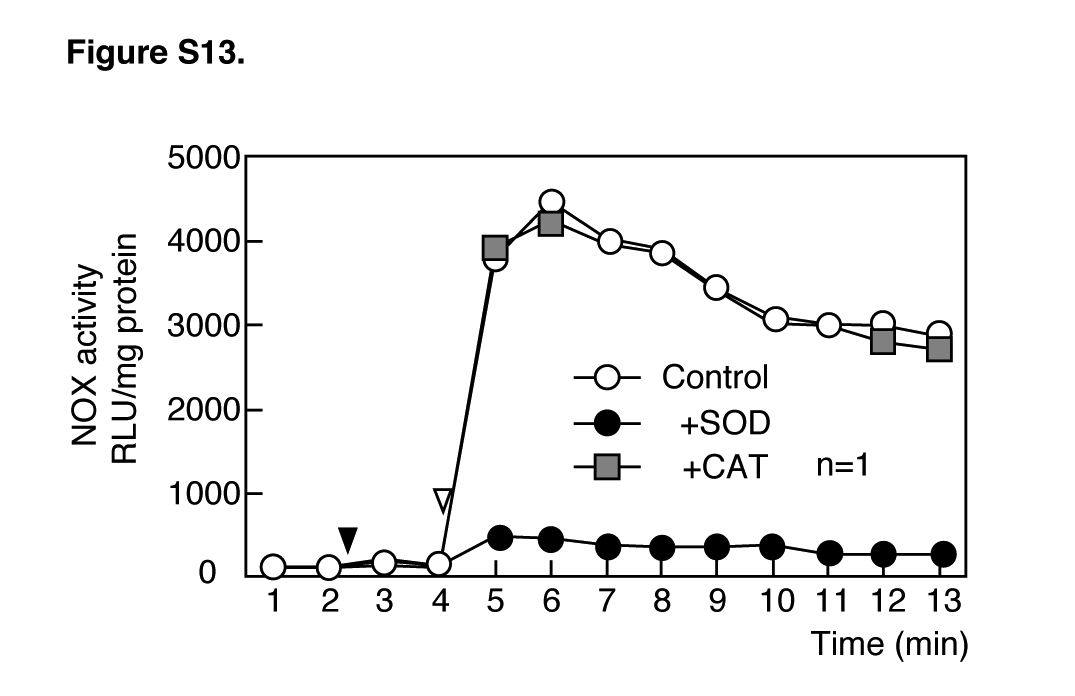
**

**Figure S13.** Control NOX activity assay. Mycelia harvested at CT 18 were disrupted and used as cell lysates in the assay. The antioxidants SOD (150 units/ml) or CAT-1 (30 µg/ml) were added to the assay buffer. Cell lysates and NADPH were added to the assay buffer at the points noted using closed and open arrows, respectively. Temporal ROS generation (RLU/mg protein) by NOX was detected using lucigenin chemiluminescence. This experiment indicates that ROS generated in this assay are superoxide radicals, but not H_2_O_2_ and that NADPH-dependent superoxide generation is due to NOX activity (see Methods S1).
