# Supplementary material for: Cross-Talk between the Cellular Redox State and the Circadian System in Neurospora
Source: PLoS One. 2011 Dec 2;6(12):e28227. doi: 10.1371/journal.pone.0028227 (PMC3229512; doi:10.1371/journal.pone.0028227)
Supplement: Figure S14 — Conidiation banding in Δnox-1 under constant darkness after 12 hr of light. The growth front was marked at 24-hr intervals (n = 12). (DOC) [file pone.0028227.s014.doc]

**
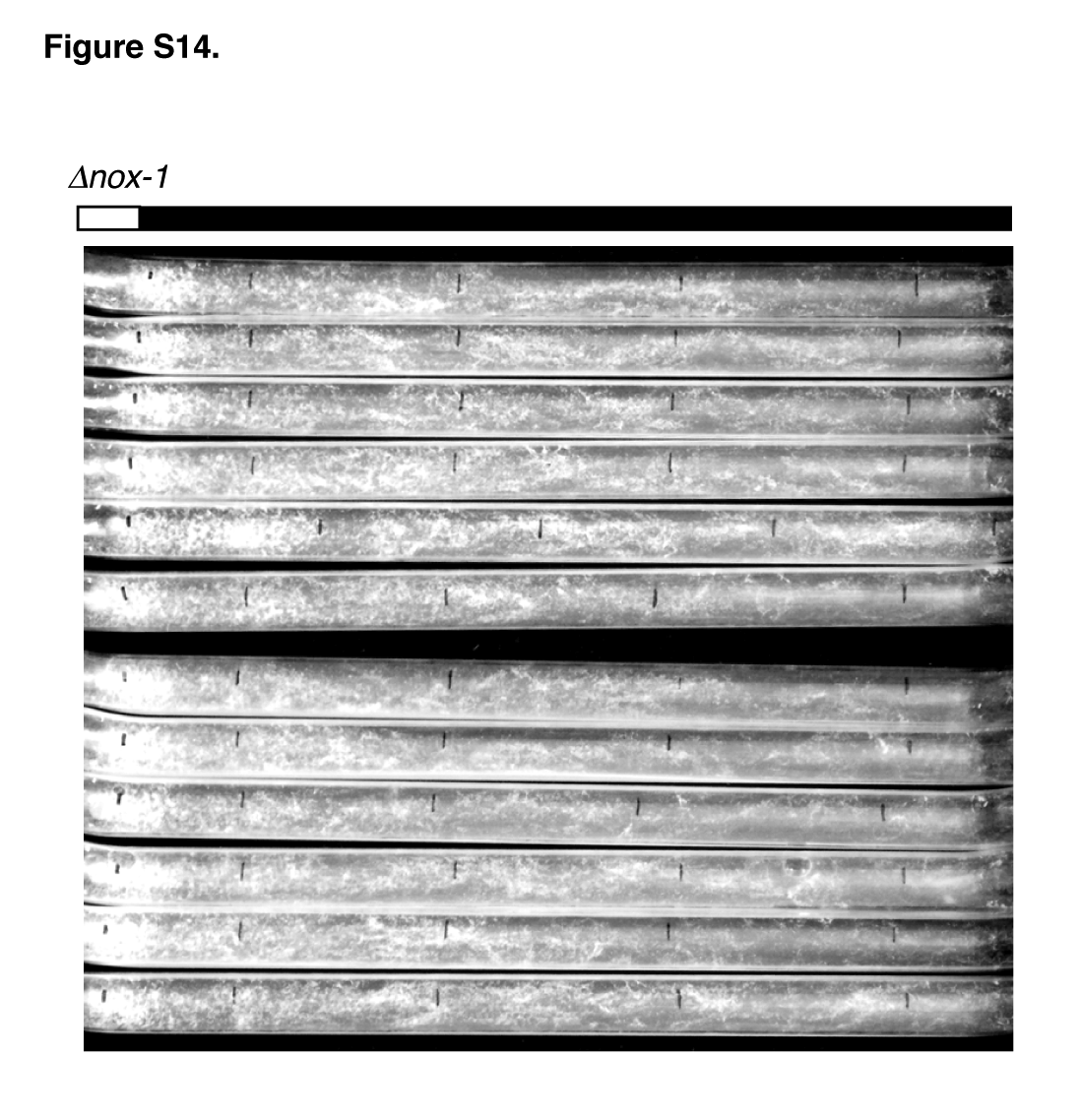
**

**Figure S14.** Conidiation banding in *∆nox-1* under constant darkness after 12 hr of light. The growth front was marked at 24-hr intervals (n=12).
