# Supplementary material for: Cross-Talk between the Cellular Redox State and the Circadian System in Neurospora
Source: PLoS One. 2011 Dec 2;6(12):e28227. doi: 10.1371/journal.pone.0028227 (PMC3229512; doi:10.1371/journal.pone.0028227)
Supplement: Figure S16 — Profiles of cellular NOX activity in Wt cells. (A) Temporal cellular NOX activity. Mycelia in the race tube growth front were harvested at CT 0, 6, 12, 18 and 24. Cellular NOX activity was measured and calculated as RLU/min/mg protein. The values shown are as relative values based on the values obtained at 11 DD. (B) Cellular NOX activity at CT 18 in subsequent cycles in Wt cells. Mycelia in the race tube growth front were harvested at CT 18 of every cycle (DD 27.5–115.5 h). Cellular NOX activities were measured and calculated as RLU/min/mg protein. The values shown are as relative values based on the values obtained at 27.5 DD. All values are shown as mean ± standard error (SEM). (DOC) [file pone.0028227.s016.doc]

**
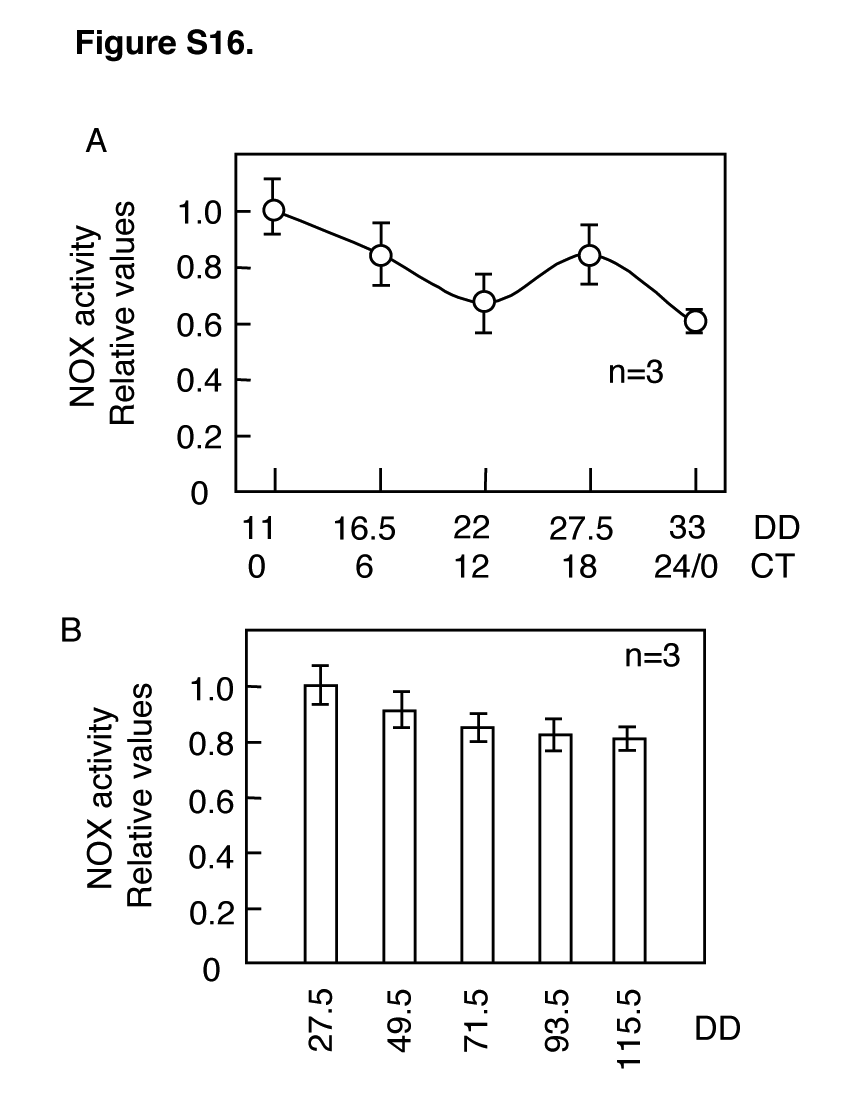
**

**Figure S16.** Profiles of cellular NOX activity in Wt cells. (A) Temporal cellular NOX activity. Mycelia in the race tube growth front were harvested at CT 0, 6, 12, 18 and 24. Cellular NOX activity was measured and calculated as RLU/min/mg protein. The values shown are as relative values based on the values obtained at 11 DD. (B) Cellular NOX activity at CT 18 in subsequent cycles in Wt cells. Mycelia in the race tube growth front were harvested at CT 18 of every cycle (DD 27.5-115.5 h). Cellular NOX activities were measured and calculated as RLU/min/mg protein. The values shown are as relative values based on the values obtained at 27.5 DD. All values are shown as mean ± standard error (SEM).
