# Supplementary material for: Cross-Talk between the Cellular Redox State and the Circadian System in Neurospora
Source: PLoS One. 2011 Dec 2;6(12):e28227. doi: 10.1371/journal.pone.0028227 (PMC3229512; doi:10.1371/journal.pone.0028227)
Supplement: Figure S17 — Control EMSA and pull-down assay. (A) EMSA control experiment using antioxidants. SOD (1,500 units/ml) or CAT-1 (75 µg/ml) was added to the reaction mixture containing 1 mM H2O2. (B) Control pull-down assay using antioxidants. SOD (1,500 units/ml) or CAT-1 (75 µg/ml) was added to the reaction mixture containing 10 mM H2O2 (see Methods S1). (DOC) [file pone.0028227.s017.doc]

**
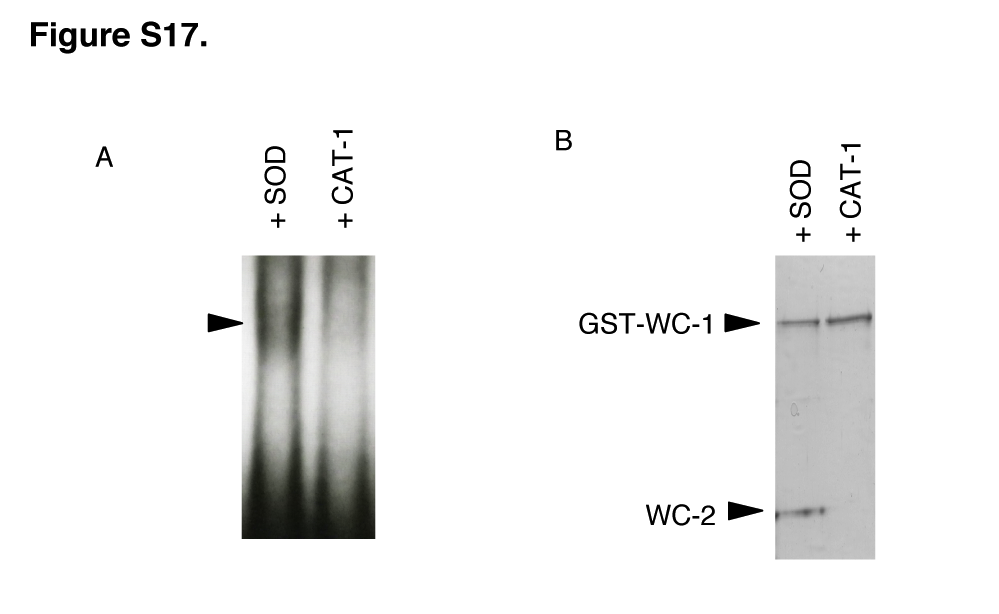
**

**Figure S17.** Control EMSA and pull-down assay. (A) EMSA control experiment using antioxidants. SOD (1,500 units/ml) or CAT-1 (75 µg/ml) was added to the reaction mixture containing 1 mM H_2_O_2_. (B) Control pull-down assay using antioxidants. SOD (1,500 units/ml) or CAT-1 (75 µg/ml) was added to the reaction mixture containing 10 mM H_2_O_2_ (see Methods S1).
