# Supplementary material for: Cross-Talk between the Cellular Redox State and the Circadian System in Neurospora
Source: PLoS One. 2011 Dec 2;6(12):e28227. doi: 10.1371/journal.pone.0028227 (PMC3229512; doi:10.1371/journal.pone.0028227)
Supplement: Figure S18 — Correlations between cellular ROS levels, frq expression and conidiation banding under free-running conditions in the Wt, cat-1RIP, sod-1 and Δnox-1. In the graph showing cellular ROS levels and frq expression, red lines indicate the levels in each strain and dotted lines indicate the Wt. (DOC) [file pone.0028227.s018.doc]

**
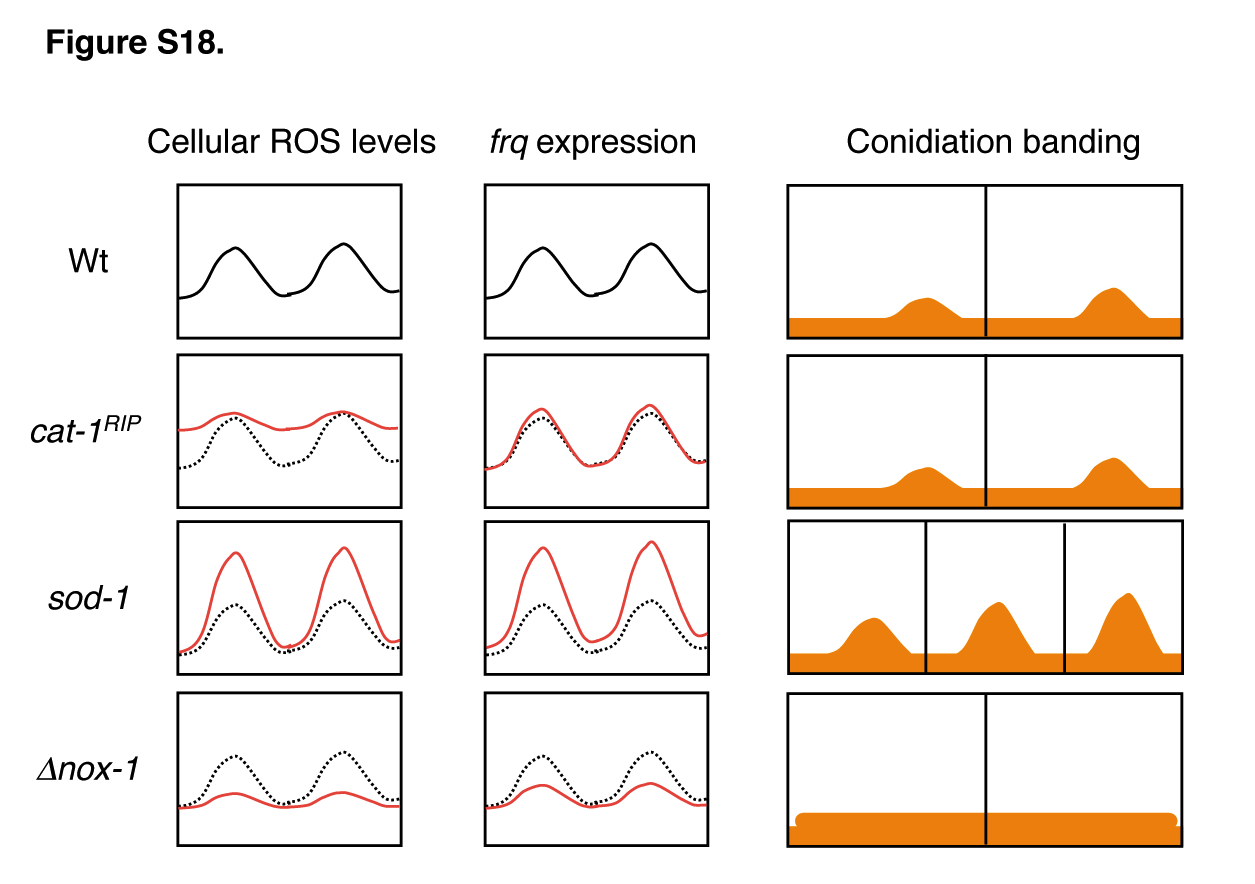
**

**Figure S18.** Correlations between cellular ROS levels, *frq* expression and conidiation banding under free-running conditions in the Wt, *cat-1^RIP^*, *sod-1* and *∆nox-1*. In the graph showing cellular ROS levels and *frq* expression, red lines indicate the levels in each strain and dotted lines indicate the Wt.
