# Supplementary material for: Cross-Talk between the Cellular Redox State and the Circadian System in Neurospora
Source: PLoS One. 2011 Dec 2;6(12):e28227. doi: 10.1371/journal.pone.0028227 (PMC3229512; doi:10.1371/journal.pone.0028227)
Supplement: Methods S1 — Materials and methods for supporting data. (DOC) [file pone.0028227.s019.doc]

Methods S1. Materials and methods for supporting data.

# ROS assay

For NBT (nitroblue tetrazolium) staining, two mycelial fragments were immersed in 1 ml of NBT solution (0.6 mM NBT and 5 mM MOPS pH 7.6) for 30 min, and the reaction was stopped by the addition of HCl (final concentration 0.05 M). The stained mycelia were observed by microscopy. Two mycelial fragments were immersed in 200 µl of Diogenes reagent (National Diagnostics, Inc., USA) for 2 min, and the dialysis membrane was removed after vortexing for 5 sec. Luminescence, assessed as relative light units (RLUs), was measured for 20 sec in a luminometer. After the measurement was taken, the mycelium was dried and weighed. Luminescence values (RLU/dry weight mg) were normalized to dry weight.

**Detection of hydrogen peroxide**

Four mycelial fragments were suspended in 0.1 M potassium phosphate buffer (pH 7.0) and sonicated for 5 sec on ice. After centrifugation, the H_2_O_2_ concentration of the lysates was quantified using the BIOXYTECH Hydrogen Peroxide Assay kit (OXIS International, Inc.). The protein concentration was determined using the CBB protein assay, and the concentration of H_2_O_2_ (µM) was normalized to the protein concentration.

# Semi-quantitative RT-PCR

The total RNA from two mycelial fragments was isolated using the RNeasy Plant Mini Kit (QIAGEN). The total RNA (1 µg) was treated with DNase I and reverse transcribed using Superscript III reverse transcriptase (Invitrogen). PCR was performed for 25 cycles with the following primers: *frq*/*for* (5-cttcctgacgaccattttgtga-3), *frq*/*rev* (5-gtcgtccccatccaccgcttct-3), *b-tub*/*for* (5-tccggcaacaagtatgtccctcgt-3), *b-tub*/*rev* (5-ggcagtgaactgctcgccgat-3).

## Antioxidants in the control assays

To evaluate specific ROS functions, in the NOX activity assay, the EMSA and the pull-down assay, we used the antioxidants SOD (from bovine erythrocytes; SIGMA) and CAT-1 (from *N. crassa*) as controls. For isolation of the recombinant catalase-1 protein, *cat-1* cDNA was cloned by RT-PCR using the following primers: *cat1-pGEX/for* (5- ccgaattctgtccaacatcatcagccaggc-3) and *cat1-pGEX/rev* (5- ccgcggccgcttagtacgcaatcatggag-3). The cDNAs were then inserted into the *Eco*RI and *Not*I sites of pGEX-6P (GE Healthcare). The *E. coli* strain BL21 was transformed with pGEX-Cat-1. Cat-1 transformants were pre-cultured in LB medium (10 % Bacto-tryptone, 5 % Bacto-yeast extract and 10% NaCl) for 24 hr at 30 ˚C. Expression of the recombinant protein was induced by 0.1 mM IPTG for 18 hr at 30 ˚C. The harvested cells were resuspended in PBS buffer (10 mM Na_2_HPO_4_, 1.8 mM KH_2_PO_4_, 140 mM NaCl, 2.7 mM KCl, 1mM DTT, and 1 mM PMSF) and lysed by sonication. Next, Triton X-100 was added to the lysate at a final concentration of 1 %, and the sample was centrifuged at 15,000 x g for 10 min at 4 ˚C. Recombinant protein was purified from the supernatant using batch purification using Glutathione Sepharose 4B (GE Healthcare). The GST tag was removed by PreScission Protease digestion for 12 hr at 4 ˚C (GE Healthcare). SOD and CAT-1 were added to the assay solution and the reaction mixture in the NOX activity assay, the EMSA, the pull-down assay and the lucigenin-induced chemiluminescence assay (see Materials and Methods).
