# Supplementary material for: Cross-Talk between the Cellular Redox State and the Circadian System in Neurospora
Source: PLoS One. 2011 Dec 2;6(12):e28227. doi: 10.1371/journal.pone.0028227 (PMC3229512; doi:10.1371/journal.pone.0028227)
Supplement: Table S1 — H2O2 concentration in Wt mycelial cells. Cellular volumes per 15,000 µm2 in mycelial fragments were determined from the microscopic images. Based on these values, the total volumes in a mycelial fragment (9×10 mm size) were calculated. H2O2 concentration in one fragment of mycelial mats was measured by BIOXYTECH Hydrogen Peroxide Assay kit. The cellular H2O2 concentrations calculated from the cellular volumes and H2O2 amounts. (DOC) [file pone.0028227.s020.doc]

**Table S1.** H_2_O_2_ concentration in Wt mycelial cells. Cellular volumes per 15,000 µm^2^ in mycelial fragments were determined from the microscopic images. Based on these values, the total volumes in a mycelial fragment (9 X 10 mm size) were calculated. H_2_O_2_ concentration in one fragment of mycelial mats was measured by BIOXYTECH Hydrogen Peroxide Assay kit. The cellular H_2_O_2_ concentrations calculated from the cellular volumes and H_2_O_2_ amounts.

|  | Cellular volumes  per 15,000 µm^2^ | Total volumes per mycelial fragment | H_2_O_2_ amounts per mycelial fragment | H_2_O_2_ concentration |
| --- | --- | --- | --- | --- |
| Wt at CT6 (DD 38.5) | 95,916 µm^3^  ±4,966 | 0.58 mm^3^  ±0.03 | 679 pmols  ±201 | 1.18 mM  ±0.35 |
| Wt at CT18 (DD 49.5) | 67,522 µm^3^  ±6,159 | 0.41 mm^3^  ±0.04 | 1,220 pmols  ±122 | 3.01 mM  ±0.30 |

n=3
